# Therapeutic effects of traditional Chinese medicine injections with heat-clearing and detoxifying properties on viral pneumonia: a systematic review and network meta-analysis

**DOI:** 10.3389/fphar.2026.1771777

**Published:** 2026-05-14

**Authors:** Yuanchong Wang, Linlin Zhao, Lu Xiao, Yihan Wang, Siyun Wang, Yifang Zhang, Changhong Miao, Xinyi Xu, Cunzhong Shi

**Affiliations:** 1 Department of Emergency, First Teaching Hospital of Tianjin University of Traditional Chinese Medicine, Tianjin, China; 2 National Clinical Research Center for Chinese Medicine, Tianjin, China; 3 Department of Oncology, First Teaching Hospital of Tianjin University of Traditional Chinese Medicine, Tianjin, China

**Keywords:** combination therapy, heat-clearing and detoxifying, network meta-analysis, traditional Chinese medicine injection, viral pneumonia

## Abstract

**Background:**

The history of traditional Chinese medicine is extensive and well-documented. Various traditional Chinese medicine injections (TCMIs) with heat-clearing and detoxifying properties, developed from classical ancient prescriptions, have been widely used in the treatment of viral pneumonia. To evaluate the efficacy and safety of various heat-clearing and detoxifying TCMIs for the treatment of viral pneumonia through a network meta-analysis.

**Methods:**

We searched eight databases to identify randomized controlled trials (RCTs) that examined heat-clearing and detoxifying TCMIs combined with conventional medicine (CM) for the treatment of viral pneumonia, published until 12 December 2025. We used R software for the Bayesian network meta-analysis.

**Results:**

Our study included 83 RCTs with a total sample size of 8,678 participants, representing seven different types of heat-clearing and detoxifying TCMIs: Reduning Injection (RDN), Xiyanping Injection (XYP), Yanhuning Injection (YHN), Tanreqing Injection (TRQ), Xuebijing Injection (XBJ), Shuanghuanglian Injection (SHL), and Qingkailing Injection (QKL). RDN + CM was the most effective treatment for shortening cough disappearance and hospitalization time, reducing interleukin-6 level, and increasing the cluster of differentiation 4/cluster of differentiation 8 ratio. Additionally, RDN + CM also showed a significant reduction in adverse events compared to CM. QKL + CM demonstrated the best results in improving the total clinical efficacy rate, shortening antipyretic time, and decreasing the duration of lung rales. Meanwhile, YHN + CM proved most effective at alleviating asthma, while also reducing levels of tumor necrosis factor alpha and high-sensitivity C-reactive protein.

**Conclusion:**

Patients with viral pneumonia benefit significantly from the use of heat-clearing and detoxifying TCMIs along with CM. Based on patient outcomes, RDN shows considerable promise and may be the preferred choice of TCMIs for the treatment of viral pneumonia.

**Systematic Review Registration:**

https://www.crd.york.ac.uk/PROSPERO/, identifier CRD42024610013.

## 1 Introduction

Viral pneumonia is a common respiratory disease caused by viral infections. It typically presents as an inflammatory condition, beginning with an infection of the upper respiratory tract mucosa that spreads downward through the airways and ultimately affects the lung tissue (Kombe Kombe et al., 2024). Viral pneumonia has an acute onset and rapidly spreads. In recent years, the incidence of viral pneumonia has increased in both adults and children, particularly during the coronavirus disease 2019 (COVID-19) pandemic, which has significantly burdened the global health system (Tompa et al., 2021). It accounts for 24.5% of community-acquired pneumonia, with influenza pneumonia being particularly prevalent (Febbo et al., 2024; Cavallazzi and Ramirez, 2024).

Treatment of viral pneumonia usually focuses on symptomatic and supportive care aimed at improving respiratory function and reducing the inflammatory response in patients. Viruses can be treated with specialized medications, such as neuraminidase inhibitors, protease inhibitors, M2 ion channel blockers, and nucleoside analogues (Meseko et al., 2023). In some cases, glucocorticoids or human immunoglobulin may be utilized for treatment (Zhang et al., 2025). However, the enormous variety of viruses, combined with their complex pathogenic mechanisms, hinders the development of effective vaccines and antiviral drugs. Furthermore, the efficacy of antiviral drugs is sometimes limited by the treatment time frames. For example, patients with influenza may need to be treated with antiviral medications such as oseltamivir within 48 h of symptom start (Chan and Hui, 2023; Pandey et al., 2023). If treatment is delayed beyond this time frame, its effectiveness may decrease, and the risk of mortality within 30 days may increase (Tenforde et al., 2025). Moreover, oseltamivir requires time to relieve symptoms such as fever and cough (Chen et al., 2021), while other antivirals like remdesivir can cause adverse effects, including sinus bradycardia (Gubitosa et al., 2020). Consequently, clinical research is actively focused on the exploration and development of new antiviral drugs.

According to the theory of traditional Chinese medicine (TCM), the key pathogenesis of viral pneumonia lies in “heat-toxin attacking the lung” or “epidemic toxin obstructing the lung.” Therefore, clinical treatment generally follows the fundamental principle of “heat-clearing and detoxifying” (Tong et al., 2020). As an important modernized dosage form developed from traditional compound prescriptions, refined TCM injections (TCMIs) produced through advanced preparation techniques have demonstrated significant value in the comprehensive treatment of this disease. Their mechanism of action does not target a single pathogen; instead, they exert broad effects on multiple pathological aspects of virus-host interactions through multi-target, thereby achieving holistic regulation and synergistic therapeutic effects (Dai et al., 2020). A growing body of evidence has demonstrated that heat-clearing and detoxifying TCMIs can effectively alleviate respiratory symptoms, inhibit viral replication, modulate immune responses, and mitigate inflammatory reactions, thereby improving clinical outcomes in patients with viral pneumonia (Jin et al., 2021). However, direct or indirect comparisons among different heat-clearing and detoxifying TCMIs are still lacking. Furthermore, the safety profile of TCMIs has garnered significant attention, as the risks associated with their use are not yet fully understood. Therefore, it is necessary to analyze potential safety risks using evidence-based methods to enhance clinical recognition (Zhao et al., 2024). This study aimed to conduct a network meta-analysis (NMA) to assess the differences in the clinical efficacy and safety among different TCMIs, providing an evidence-based foundation for clinical medication selection in cases of viral pneumonia.

## 2 Materials and methods

### 2.1 Study registration

This systematic review was registered on the PROSPERO platform under registration number CRD42024610013. The conduct and reporting of the study followed the Preferred Reporting Items for Systematic Reviews and Meta-Analysis (PRISMA) statement (Page et al., 2021). Further information can be found in Supplementary Material S1.

### 2.2 Study section criteria

The PICOTS framework was employed to define the study scope. Population (P): Patients diagnosed with viral pneumonia, regardless of age, sex, or disease severity, according to established diagnostic criteria (Qu and Cao, 2016; Lu et al., 2019). Intervention (I): Adjunctive therapy with any heat-clearing and detoxifying TCMIs administered in combination with conventional medicine (CM). Comparator (C): CM alone. This encompassed antiviral agents, antipyretics, antitussives, expectorants, oxygen therapy, glucocorticoids when indicated, intravenous immunoglobulins, and management of secondary infections. Outcomes (O): The primary outcomes included total clinical effective rate, antipyretic time, cough disappearance time, tumor necrosis factor alpha (TNF-α) level, interleukin (IL)-6 level, and incidence of adverse reactions. Secondary outcomes included disappearance time of lung rales, duration of asthma, hospitalization time, cluster of differentiation (CD) 4 level, CD4/CD8 ratio, Immunoglobulin (Ig) M level, IgG level, IL-8 level, and high-sensitivity C-reactive protein (hs-CRP) level. Time (T): No specific time limit was set. Study Design (S): Only randomized controlled trials (RCTs) published in English or Chinese were considered for inclusion.

Exclusion criteria were as follows: reviews, animal or cell experiments, missing original data, repeated publication of literature, meta-analysis, intervention measures of the control group combined with TCMIs, the diagnosis of the patient was not clear and could not be identified as viral pneumonia, and the full text of the study could not be obtained.

### 2.3 Search strategy

We systematically retrieved journal articles on the treatment of viral pneumonia using TCMIs from eight databases. These included four English databases: PubMed, Web of Science (WOS), Cochrane Library, and Embase; and four Chinese databases: the China National Knowledge Infrastructure (CNKI), WanFang, Chinese Biomedical Literature Database (CBM), and China Science and Technology Journal Database (VIP). The search was conducted for materials published from the inception of each database until 12 December 2025. Our search strategy integrated both subject terms and free-text keywords, such as “viral pneumonia,” “traditional Chinese medicine injection,” “injection,” “extract,” and “traditional Chinese medicine.” We also tailored our approach to fit the unique characteristics of each database to ensure a comprehensive and relevant collection of search results (Supplementary Material S2).

### 2.4 Literature screening and data extraction

Two researchers (YC Wang and LL Zhao) independently screened the literature, extracted data according to the inclusion and exclusion criteria, and consulted with a third researcher (L Xiao) when they encountered differences. The extracted data included the main author, publication year, general condition of the patients, intervention measures, outcome indicators, and the course of treatment. Moreover, missing values in the data were treated with appropriate methods, giving priority to the assignment of dummy variables, and then considering the replacement with mean values or medians.

### 2.5 Literature quality evaluation

We evaluated the quality of the literature using the Cochrane risk of bias (RoB) 2.0 tool (Sterne et al., 2019). The evaluation encompassed five aspects: randomization process, deviation from established interventions, integrity of outcome data, measurement of outcome, and selective reporting of results. Each aspect was evaluated on three levels: “high risk,” “low risk,” and “certain risk.” Two researchers (YC Wang and LL Zhao) independently performed a literature quality evaluation according to these criteria. In case of any disagreement, a consensus was first reached through consultation among the researchers, and if academic disputes remained, a third researcher (L Xiao) was invited to intervene in arbitration. Furthermore, the Grading of Recommendations, Assessment, Development and Evaluation (GRADE) tool was utilized to assess the degree of certainty in the evidence, offering an evaluation of the confidence level (Brozek et al., 2009).

### 2.6 Statistically analysis

We used the R software (version 4.4.2) for statistical analysis and performed NMA with the BUGSnet, GeMTC, and netmeta packages. The Bayesian model constructed in this study was selected based on the deviance information criterion (DIC) results, where a lower DIC value indicates better model fit (Béliveau et al., 2019). Heterogeneity across studies was assessed using the I^2^ statistic. The network graph was constructed to illustrate direct comparisons among interventions. Consistency was evaluated via the node splitting method. For evidence structures that did not form closed loops, the consistency assumption was not applicable. In the fitted model, we set n.chain = 4, thin = 1, n.adapt = 20,000, and n.iter = 50,000. Outcome measures were expressed as risk ratios (RR) for dichotomous variables and mean differences (MD) for continuous variables, each reported with a 95% confidence interval (CI). Treatment rankings for each outcome were generated using the surface under the cumulative ranking curve (SUCRA) and rankograms, with heatmaps created based on ranking results. The performance of interventions was further compared using forest plots. Finally, Egger’s test was applied to produce a comparison-adjusted funnel plot. According to Cochrane guidelines, if the number of included studies is less than ten, no statistical test will be conducted to examine the asymmetry of the funnel plot.

### 2.7 Sensitivity analysis

Sensitivity analyses used a leave-one-out approach with the GeMTC package in R software (version 4.4.2). In this procedure, each study was sequentially omitted, and the NMA was repeated to test if any single study had a substantial influence on the overall effect estimates.

### 2.8 Subgroup analysis

Subgroup analyses were performed using the meta package in R software (version 4.4.2) to explore potential sources of heterogeneity. Studies were stratified by age into children (<18 years) and adults (≥18 years). In addition, subgroup analyses were planned according to treatment duration (<7 days and ≥7 days) when sufficient data were available. Studies with unreported treatment duration were to be considered as a separate subgroup, where applicable.

## 3 Results

### 3.1 Literature search

**FIGURE 1:**
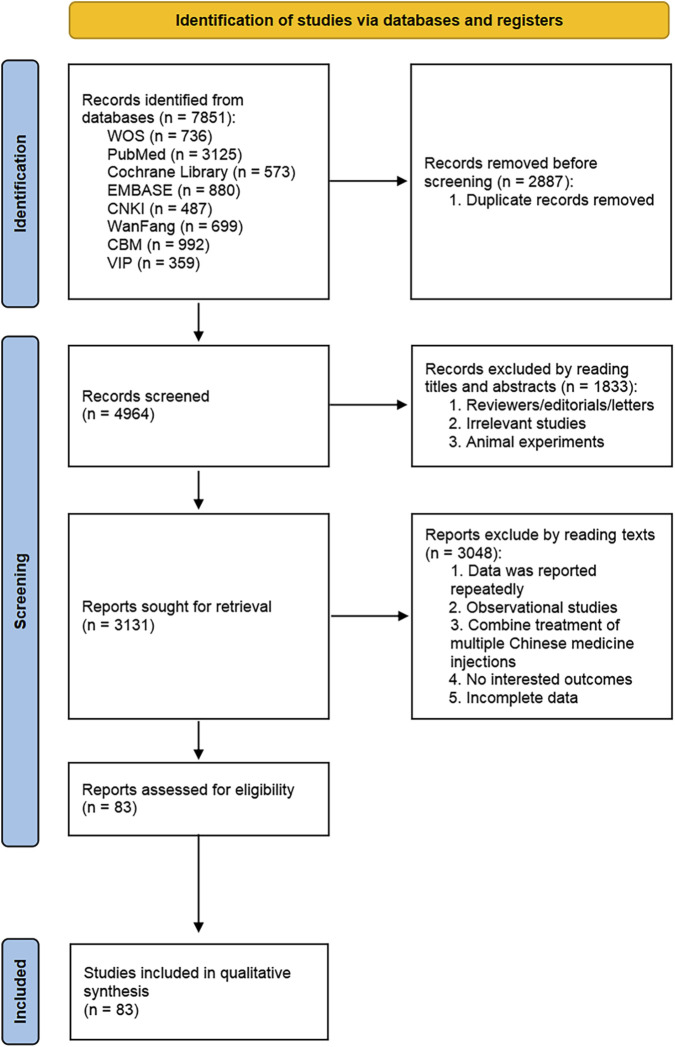
Literature search process.

A total of 7,851 articles were retrieved, with 2,887 duplicates eliminated. After reviewing the titles and abstracts, we eliminated 1,833 animal experiments, reviews, and articles not pertinent to the topic. After screening, we read the full text and included 83 articles. These comprised seven types of heat-clearing and detoxifying TCMIs: Reduning Injection (RDN), Xiyanping Injection (XYP), Yanhuning Injection (YHN), Tanreqing Injection (TRQ), Xuebijing Injection (XBJ), Shuanghuanglian Injection (SHL), and Qingkailing Injection (QKL). The screening process is displayed in Figure 1. Moreover, Supplementary Material S5 provided additional details, including the metabolites of the heat-clearing and detoxifying TCMIs. We employed the ConPhYMP tool to assess the composition of the formulation and the guidelines for the processing procedure, as detailed in Supplementary Material S7 (Heinrich et al., 2022).

### 3.2 Basic characteristics of included studies and quality evaluation

**FIGURE 2:**
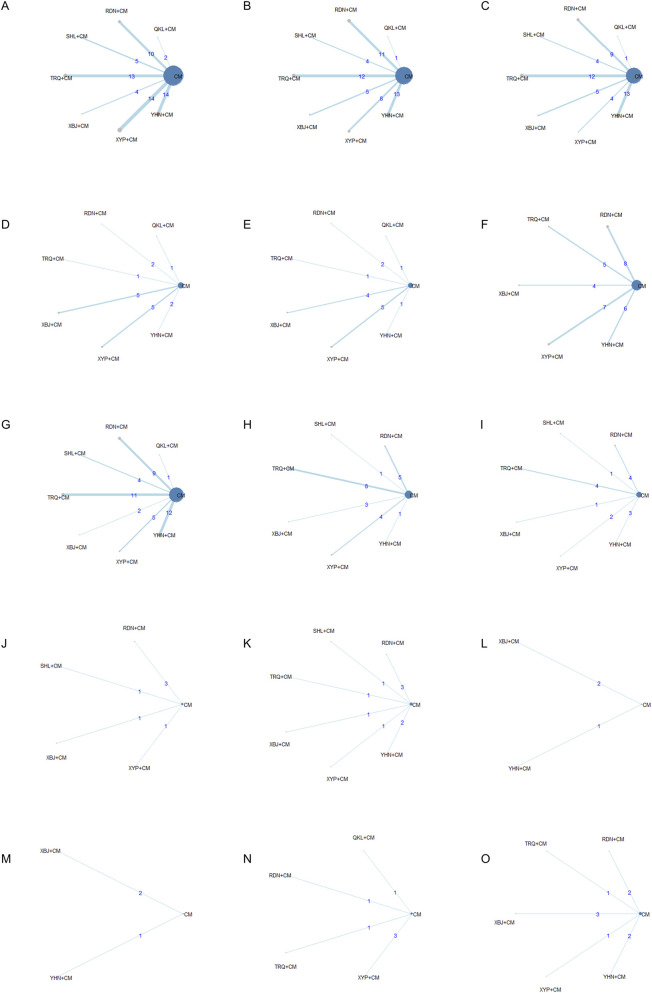
Network graph. **(A)** Total clinical effective rate; **(B)** Antipyretic time; **(C)** Cough disappearance time; **(D)** TNF-α level; **(E)** IL-6 level; **(F)** Incidence of adverse reactions; **(G)** Disappearance time of lung rales; **(H)** Duration of asthma; **(I)** Hospitalization time; **(J)** CD4 level; **(K)** CD4/CD8 ratio; **(L)** IgM level; **(M)** IgG level; **(N)** IL-8 level; **(O)** hs-CRP level.

**FIGURE 3:**
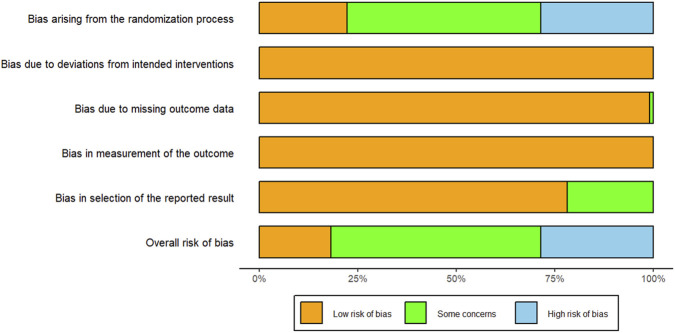
Percentages of items of included articles that produced risks of bias.

We analyzed 83 RCTs completed between 2000 and 2025, including 17 RDN, 18 XYP, 17 YHN, 16 TRQ, 7 XBJ, 6 SHL, and 2 QKL studies. A total of 8,678 participants were enrolled: 4,382 in the experimental group and 4,296 in the control group. Table 1, Figure 2, and Supplementary Material S3 provide basic information about the listed study. Since no closed loops were formed in the evidence network for the outcome indicators, inconsistency tests were not conducted. Isolated nodes or imbalanced connections in the network could suggest violations of the transitivity assumption; these were further examined using box plots (Supplementary Material S4). Among the 83 studies, 65 used a randomized approach. Specifically, 18 studies employed a random number table approach, 2 studies used simple randomization, and 45 studies simply declared “random” without mentioning the allocation method. Furthermore, six trials grouped participants according to treatment schedule or admission order, a method that does not constitute randomization. The risk of bias is shown in Figure 3.

**TABLE 1:** Basic characteristics of included literature.

Study	Sample size		Sex		Age (years)	Treatment		Course (days)	Clinical outcomes
	T	C	M	F		T	C		
Zou and Wang (2000)	40	40	48	32	0.10 ∼ 1.5	SHL 10 ∼ 15 mL·kg^-1^·d^-1^ Qd + CM	CM	NA	b, c, g, i
Qin and Wang (2003)	30	30	34	26	0.5 ∼ 4	SHL 0.6 mg Bid + CM	CM	5 ∼ 7	a, b, c, g, h
Hu and Zhu (2006)	38	34	46	26	T: 20 ± 10 C: 24 ± 10	TRQ 20 mL Qd + CM	CM (antiviral drugs and so on)	10	a
Yang (2007)	60	56	62	54	T: 0.5 ∼ 8 C: 0.67 ∼ 7	YHN 10 mg·kg^-1^·d^-1^ Qd + CM	CM (antiviral drugs and so on)	7 ∼ 14	a, b, c, g
Huang (2008)	30	30	31	29	<18	YHN 5 ∼ 10 mg·kg^-1^·d^-1^ + CM	CM (antiviral drugs and so on)	NA	b, c, g, h, i
Wang et al. (2008)	60	57	55	62	T: ≤6 C: ≤11	YHN 5 ∼ 15 mg·kg^-1^·d^-1^ Qd + CM	CM (antiviral drugs and so on)	7	a, b, c, g, i
Guo (2009)	40	40	37	43	T: 0.17 ∼ 11 C: 0.25 ∼ 12	XYP 5 ∼ 10 mg Qd + CM	CM (antiviral drugs and so on)	5 ∼ 7	b, g, h, i
Li (2010)	100	100	114	86	T: 1.125 C: 1.175	YHN 10 mg·kg^-1^·d^-1^ Qd + CM	CM (glucocorticoids and so on)	7	a
Liu et al. (2010a)	72	58	61	69	T: 0.67 ∼ 7 C: 0.75 ∼ 7	QKL 10 ∼ 20 mL Qd + CM	CM (antiviral drugs and so on)	NA	a
Liu et al. (2010b)	50	50	53	47	T: 0.75 ∼ 6 C: 0.83 ∼ 6	YHN 80 ∼ 160 mg Qd + CM	CM (antiviral drugs and so on)	NA	a, b, c, g
Wang (2010)	85	85	103	67	T: 41.3 C: 42	SHL 2.4 g·d^-1^ + CM	CM (antiviral drugs and so on)	NA	a
Li (2011)	50	50	55	45	T: 0.75 ∼ 5.5 C: 0.67 ∼ 5	YHN 80 ∼ 160 mg Qd + CM	CM (antiviral drugs and so on)	NA	a
Xiang et al. (2011)	46	40	41	45	T: 4.03 ± 1.17 C: 4.07 ± 1.12	RDN 0.6 mL·kg^-1^·d^-1^ + CM	CM	5 ∼ 7	a, b, g
Ye and Su (2011)	30	30	NA	NA	0.83 ∼ 7	TRQ 0.3 ∼ 0.5 mL·kg^-1^·d^-1^ Qd + CM	CM (antiviral drugs and so on)	7	b, c, g, i
Cao and Xue (2012)	34	34	48	20	T: 48.3 ± 8.7 C: 47.2 ± 7.9	TRQ 20 mL Qd + CM	CM (antiviral drugs and so on)	14	a, b
Cheng (2012)	120	120	104	136	4 ∼ 8	SHL 30 mg·kg^-1^·d^-1^ Qd + CM	CM (antiviral drugs and so on)	NA	a, b, c, g
Gao (2012)	78	78	96	60	0.33 ∼ 3	XYP 0.4 mL·kg^-1^·d^-1^ + CM	CM (antiviral drugs and so on)	5 ∼ 7	a
Liu et al. (2012)	67	53	58	62	T: 0.75 ∼ 7 C: 0.67 ∼ 7	YHN 5 ∼ 10 mg·kg^-1^·d^-1^ Qd + CM	CM (antiviral drugs and so on)	5 ∼ 7	a, b, c, g, f
Wang and Jin (2012)	61	61	63	59	T: 2.98 ± 0.61 C: 2.52 ± 1.82	XYP 5 ∼ 10 mg·kg^-1^·d^-1^ + CM	CM (antiviral drugs and so on)	5 ∼ 7	a, b, g, h
Yang (2012)	43	43	45	41	T: 41.0 ± 2.2 C: 39.0 ± 3.6	SHL 10 mL·kg^-1^ Qd + CM	CM (antiviral drugs and so on)	5	a
Jiang (2013)	50	50	53	47	T:0.75 ∼ 6 C: 0.83 ∼ 6	YHN 80 ∼ 160 mg Qd + CM	CM (antiviral drugs and so on)	NA	a, b, c, g
Li and Wu (2013)	32	32	38	26	57.52 ± 12.90	XYP 30 mL Qd + CM	CM	7	a, b
Li (2013)	63	35	59	39	3.1 ± 0.4	YHN 20 ∼ 160 mg Qd + CM	CM (antiviral drugs and so on)	7 ∼ 10	a, b, c, g
Qing (2013)	45	45	57	33	T: 5.1 ± 1.2 C: 4.8 ± 1.1	SHL 30 mg·kg^-1^·d^-1^ Qd + CM	CM (antiviral drugs and so on)	7	a, b, c, g, j, k
Shi and Yan (2013)	25	25	23	27	T: 0.75 ∼ 8 C: 0.67 ∼ 10	YHN 10 mg·kg^-1^·d^-1^ Qd + CM	CM (antiviral drugs and so on)	7	a, b, c, g
Zhao (2013)	40	40	51	29	T: 4.26 ± 1.37 C: 4.13 ± 1.28	TRQ 0.3 ∼ 0.5 mL·kg^-1^ Qd + CM	CM (antiviral drugs and so on)	7	a, b, c, g
Kang (2015)	126	108	NA	NA	T: 18 ∼ 45 C: 20 ∼ 50	XYP 3 mL Qd + CM	CM (antiviral drugs and so on)	7	a
Lin (2015)	36	30	44	22	36.6 ± 5.4	RDN 0.6 mL·kg^-1^·d^-1^ Qd + CM	CM (immunoglobulin and so on)	5	b, c, g, h, j, k, f
Xue (2015)	38	38	42	34	T: 0.17 ∼ 2.33 C: 0.25 ∼ 2.17	TRQ 0.3 mL·kg^-1^ Qd + CM	CM (antiviral drugs and so on)	NA	b, c, g, h, f
Zhang and Zhou (2015)	153	152	161	144	T: 2.3 ± 0.8 C: 2.4 ± 0.9	XYP 10 mg·kg^-1^·d^-1^ Qd + CM	CM (antiviral drugs and so on)	7	a
Luo (2016)	45	45	53	37	T: 0.42 ∼ 1.08 C: 0.5 ∼ 1	TRQ 0.3 ∼ 0.5 mL·kg^-1^ Qd + CM	CM (antiviral drugs and so on)	NA	a, c, g, h, i, f
Wang et al. (2016)	60	60	NA	NA	<18	XYP 5 mg·kg^-1^·d^-1^ + CM	CM (antiviral drugs and so on)	7	a, b, c, g, d, e, n, f
Wu et al. (2016)	46	46	53	40	54.24 ± 10.22	XBJ 50 mL Bid + CM	CM (immunoglobulin and so on)	7	a, b, c, h, d, e, o
Xiao (2016)	50	50	53	47	T: 0.5 ∼ 3.17 C: 0.58 ∼ 3.33	XYP 5 mg·kg^-1^·d^-1^ + CM	CM (immunoglobulin and so on)	3 ∼ 5	a
Yin et al. (2016)	34	33	NA	NA	T: 57.9 ± 4.3 C: 59.2 ± 4.6	TRQ 20 mL Bid + CM	CM (antiviral drugs and so on)	14	a, b, c, h
Zhao et al. (2016)	50	50	62	38	T: 3.3 ± 1.1 C: 3.5 ± 1.2	RDN 0.6 mL·kg^-1^·d^-1^ Qd + CM	CM (immunoglobulin and so on)	5	a, b, c, g, j, o, f
Chen (2017)	53	53	55	51	T: 2.9 ± 1.3 C: 3.0 ± 1.2	YHN 10 mg Qd + CM	CM (antiviral drugs and so on)	7	a, b, c, g
Guo (2017)	32	32	35	29	T: 3.4 ± 2.2 C: 3.8 ± 2.1	XYP 0.3 mg·kg^-1^·d^-1^ + CM	CM (antiviral drugs and so on)	10	a
Li et al. (2017)	53	52	62	43	T: 3.05 ± 0.83 C: 3.14 ± 0.79	QKL 6 ∼ 10 mL Qd + CM	CM (antiviral drugs and so on)	7	a, b, c, g, d, e, n
Li (2017)	35	35	NA	NA	T: 6.88 ± 1.46 C: 7.12 ± 1.34	XYP 10 mg·kg^-1^·d^-1^ Qd + CM	CM (antiviral drugs and so on)	7	a
Liu et al. (2017)	60	68	75	53	T: 26.2 ± 8.7 C: 25.8 ± 9.1	XYP 5 ∼ 10 mg·kg^-1^·d^-1^ Qd + CM	CM (antiviral drugs and so on)	7	a, f
Luo (2017)	47	45	43	49	T: 7.51 ± 3.52 C: 7.42 ± 3.56	RDN 20 mL·d^-1^ + CM	CM (antiviral drugs and so on)	5	b, i, f
Zeng (2018)	31	31	43	19	T: 3.4 ± 0.3 C: 3.5 ± 0.9	RDN 0.6 mL·kg^-1^ Qd + CM	CM (antiviral drugs and so on)	5	a, b, c, g, h, f
Zhai (2018)	44	44	54	34	T: 4.5 ± 2.5 C: 4.2 ± 2.6	RDN 0.6 mL·kg^-1^·d^-1^ Qd + CM	CM (antiviral drugs and so on)	5	j, k
Gao C. et al. (2018)	61	61	66	56	T: 5.5 ± 1.7 C: 5.7 ± 1.6	XYP 5 ∼ 10 mg·kg^-1^ Qd + CM	CM (antiviral drugs and so on)	21	a, d, e, f
Jing (2018)	150	150	152	148	T: 47.5 ± 1.4 C: 47.2 ± 1.6	XBJ 50 mL Tid + CM	CM (immunoglobulin and so on)	30	d, e, o
Liang (2018)	39	39	41	37	T: 0.17 ∼ 1.92 C: 0.08 ∼ 1.83	TRQ 0.4 mL·kg^-1^ Qd + CM	CM (antiviral drugs and so on)	7	a, b, c, g, h, f
Liu (2018)	98	98	101	95	T: 2.3 ± 1.5 C: 2.4 ± 1.5	TRQ 0.3 ∼ 0.5 mg·kg^-1^ Qd + CM	CM (antiviral drugs and so on)	7	a, b, c, g, h, i, f
Song and Yang (2018)	60	60	62	58	T: 1.62 ± 0.46 C: 1.54 ± 0.41	XYP 0.2 ∼ 0.4 mL·kg^-1^·d^-1^ + CM	CM	NA	d, e, n
Yu (2018)	31	31	33	29	T: 5.4 ± 1.7 C: 5.5 ± 1.3	RDN 20 mL·d^-1^ + CM	CM (antiviral drugs and so on)	5	a
Zhang (2018)	50	50	55	45	T: 50 ∼ 69 C: 49 ∼ 68	TRQ 20 mL Bid + CM	CM (antiviral drugs and so on)	7	a, b, c, g, h
Zhang et al. (2018)	50	52	47	55	<18	XYP 2.5 mg·kg^-1^ Tid + CM	CM (glucocorticoids and so on)	7	a, c, h, f
Zhao et al. (2018)	70	70	69	71	T: 5.78 ± 1.67 C: 5.05 ± 1.34	RDN ≤10 mL·d^-1^ Qd + CM	CM (antiviral drugs and so on)	5 ∼ 7	a, d, n
Wang and Liu (2019)	44	44	47	41	T: 0.5 ∼ 1 C: 0.5 ∼ 1.08	TRQ 0.3 ∼ 0.5 mL·kg^-1^ Qd + CM	CM (antiviral drugs and so on)	7	a
Zhang (2019)	120	120	131	109	T: 57.51 ± 4.13 C: 58.04 ± 4.89	TRQ 20 mL Bid + CM	CM (antiviral drugs and so on)	NA	a, b, c, g, h
Fan et al. (2020)	80	80	78	82	T: 4.79 ± 0.53 C: 4.81 ± 0.46	YHN 0.4 g·d^-1^ + CM	CM (antiviral drugs and so on)	5	a, k, f
Jiang and Zuo (2020)	30	30	33	27	T: 5.32 ± 0.17 C: 4.58 ± 0.29	RDN 0.6 mL·kg^-1^·d^-1^ Qd + CM	CM (immunoglobulin and so on)	NA	b, c, g
Lan and Yan (2020)	67	67	65	59	T: 1.35 ± 0.65 C: 1.42 ± 0.37	RDN 0.6 mL·kg^-1^ + CM	CM (antiviral drugs and so on)	7	a, b, c, g, h
Li and Pan (2020)	51	51	57	45	4.68 ± 2.51	YHN 160 ∼ 400 mg Qd + CM	CM (antiviral drugs and so on)	6	b, c, g
Li and Zhang (2020)	60	60	69	51	T: 50.00 ± 3.50 C: 49.50 ± 3.00	XBJ 50 mL Bid + CM	CM (immunoglobulin and so on)	7	a, b, c, g, d, l, m, f
Lin et al. (2020)	34	34	32	36	T: 3.43 ± 1.87 C: 3.24 ± 1.80	YHN 0.16 ∼ 0.4 g·d^-1^ Qd + CM	CM (antiviral drugs and so on)	7	a, b, c, g, d, e, f
Qiao and He (2020)	55	55	63	47	T: 4.69 ± 0.28 C: 4.71 ± 0.35	TRQ 0.5 mL·kg^-1^ Qd + CM	CM (antiviral drugs and so on)	14	a, b, c, g, i, d, e, n, o
Qin et al. (2020)	21	26	23	24	T: 58.0 ± 2.9 C: 58.3 ± 2.9	RDN 20 mL Qd + CM	CM (glucocorticoids and so on)	5 ∼ 7	i, k, e
Zhang et al. (2020)	22	22	22	22	T: 49.05 ± 14.19 C: 45.95 ± 14.68	XBJ 100 mL Bid + CM	CM (antiviral drugs and so on)	7	a, f
Hu et al. (2021)	49	48	52	45	T: 4.52 ± 1.23 C: 4.13 ± 1.58	YHN 10 mg·kg^-1^ Qd + CM	CM (antiviral drugs and so on)	5	k, o, f
Li and Xiong (2021)	27	27	30	24	T: 3.39 ± 1.94 C: 3.24 ± 1.80	RDN 0.5 ∼ 0.6 mL·kg^-1^·d^-1^ (≤10 mL) Qd + CM	CM (antiviral drugs and so on)	7	a, b, c, g
Su (2021)	57	56	60	53	T: 53.1 ± 6.9 C: 53.7 ± 6.5	XBJ 50 mL Bid + CM	CM (immunoglobulin and so on)	7	b, c, h, d, l, m, e, o
Xu (2021)	56	56	69	43	T: 6.21 ± 0.22 C: 6.13 ± 0.40	TRQ 0.5 mL·kg^-1^ Qd + CM	CM (antiviral drugs and so on)	14	a, b, c, g
Zuo (2021)	50	50	50	50	4.43 ± 1.28	RDN 0.2 ∼ 0.4 mL·kg^-1^·d^-1^ + CM	CM (antiviral drugs and so on)	5	a, f
Zhang et al. (2021)	65	65	32	28	T: 44.31 ± 13.45 C: 48.25 ± 14.22	XYP 10 mg·kg^-1^ Qd + CM	CM (antiviral drugs and so on)	7 ∼ 14	b
Gao et al. (2022)	43	43	48	38	T: 51.51 ± 5.73 C: 52.52 ± 6.08	XBJ 50 mL Bid + CM	CM (antiviral drugs and so on)	7	b, c, g, h, d, j, k, f
Luo et al. (2022)	45	45	51	39	T: 5.80 ± 1.46 C: 5.49 ± 1.22	RDN 0.5 mL·kg^-1^ Qd + CM	CM (antiviral drugs and so on)	7	a, b, c, g, i, d, f
Mo et al. (2022)	41	41	53	39	T: 42.56 ± 7.83 C: 44.61 ± 5.96	XYP 0.5 mL·kg^-1^ Qd + CM	CM (antiviral drugs and so on)	7	a, b, g, h, d, j, k, e, n, f
Chang (2023)	41	41	41	41	T: 3.21 ± 0.40 C: 3.02 ± 0.45	YHN 5 ∼ 10 mg·kg^-1^·d^-1^ Qd + CM	CM (antiviral drugs and so on)	7	a, b, c, g, i, l, m, o, f
Gao and Du (2023)	49	49	51	47	T: 45.65 ± 5.56 C: 45.65 ± 5.56	RDN 20 mL Qd + CM	CM (antiviral drugs and so on)	5	b, c, h, e, o, f
Guo and Huang (2023)	20	20	27	13	T: 65 C: 63	TRQ 30 mL Qd + CM	CM	7	k
Zhang et al. (2023)	36	41	46	31	T: 83.25 ± 12.22 C: 79.43 ± 13.12	RDN 20 mL Qd + CM	CM (antiviral drugs, glucocorticoids, and so on)	7	i
Li (2024)	51	51	53	29	T: 45.08 ± 5.46 C: 45.62 ± 6.06	RDN 20 mL Qd + CM	CM (antiviral drugs and so on)	5	a, b, c, g, h, f
Liu (2024)	30	30	33	27	T: 34.53 ± 10.21 C: 33.59 ± 9.97	XYP 500 mg Qd + CM	CM (antiviral drugs and so on)	5	b, c, f
Wang et al. (2024)	38	36	42	32	T: 65.53 ± 8.04 C: 64.21 ± 8.02	XBJ 50 mL Bid + CM	CM (antiviral drugs and so on)	14	a, b, c, i, e, f
Mao et al. (2024)	40	40	44	36	T: 42.50 ± 3.32 C: 42.28 ± 3.62	YHN 400 mg Qd + CM	CM (antiviral drugs and so on)	5	a, b, c, d, f
Cai (2025)	39	39	44	34	T: 3.19 ± 0.10 C:3.22 ± 0.14	XYP 5 ∼ 10 mg·kg^-1^ Qd + CM	CM (antiviral drugs and so on)	5	a, b, c, g, i, d, e, o, f
Yang et al. (2025)	30	30	33	27	T:38.71 ± 3.21 C: 38.67 ± 3.18	TRQ 20 mL Qd + CM	CM (antiviral drugs and so on)	5	a, b, c, g, h, f

T: treatment; C: control; M: male; F: female; CM: conventional medicine; a: Total clinical effective rate; b: Antipyretic time; c: Cough disappearance time; d: TNF-α level; e: IL-6 level; f: Incidence of adverse reactions; g: Disappearance time of lung rales; h: Duration of asthma; i: Hospitalization time; j: CD4 level; k: CD4/CD8 ratio; l: IgM level; m: IgG level; n: IL-8 level; o: hs-CRP level.

### 3.3 Certainty of the evidence

All evidence was evaluated using the GRADE tool. The findings indicate that the confidence of the predicted values for all outcomes ranged from low to high (Supplementary Material S6).

### 3.4 Effect model selection

We compared the effect model for each outcome indicator based on the size of the DIC and I^2^. At the same time, as all of the results in the NMA are non-closed loops, the consistency assumption did not apply to this study. The fixed effects model was selected for the total clinical effective rate and incidence of adverse reactions of the outcome variables. In contrast, the random effects model was selected for the antipyretic time, cough disappearance time, TNF-α level, IL-6 level, disappearance time of lung rales, duration of asthma, hospitalization time, CD4 level, CD4/CD8 ratio, IgM level, IgG level, IL-8 level, and hs-CRP level. With I^2^ values below 50% for the best-fitting model across all outcomes, the heterogeneity was deemed acceptable. More details of DIC and I^2^ can be found in Supplementary Material S4.

## 4 Outcomes

### 4.1 Primary outcomes

#### 4.1.1 Total clinical effective rate

**FIGURE 4:**
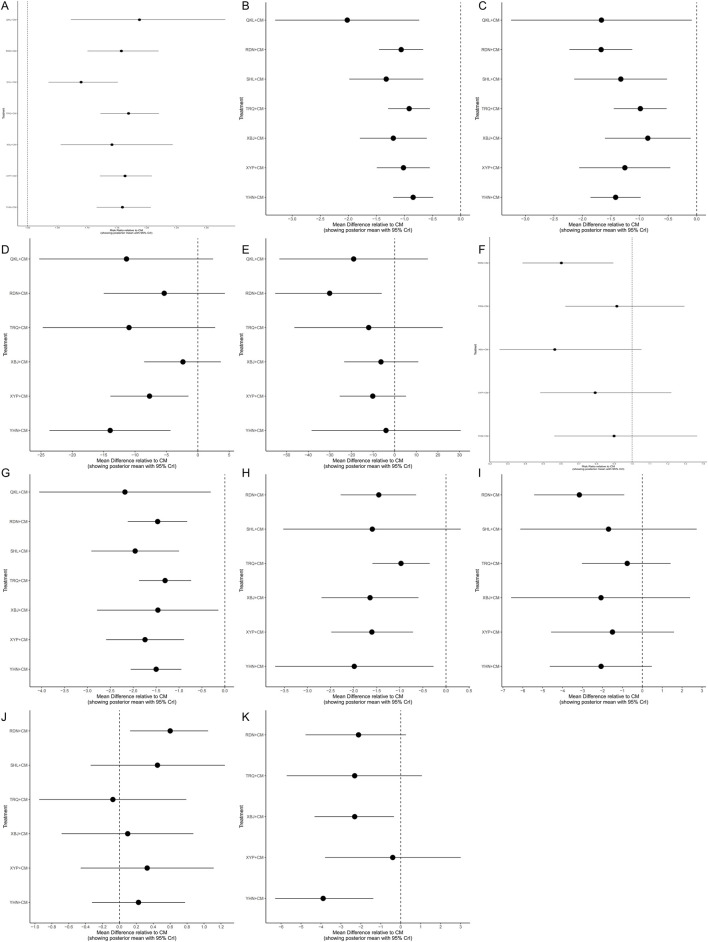
Forest plot. **(A)** Total clinical effective rate; **(B)** Antipyretic time; **(C)** Cough disappearance time; **(D)** TNF-α level; **(E)** IL-6 level; **(F)** Incidence of adverse reactions; **(G)** Disappearance time of lung rales; **(H)** Duration of asthma; **(I)** Hospitalization time; **(J)** CD4/CD8 ratio; **(K)** hs-CRP level.

**FIGURE 5:**
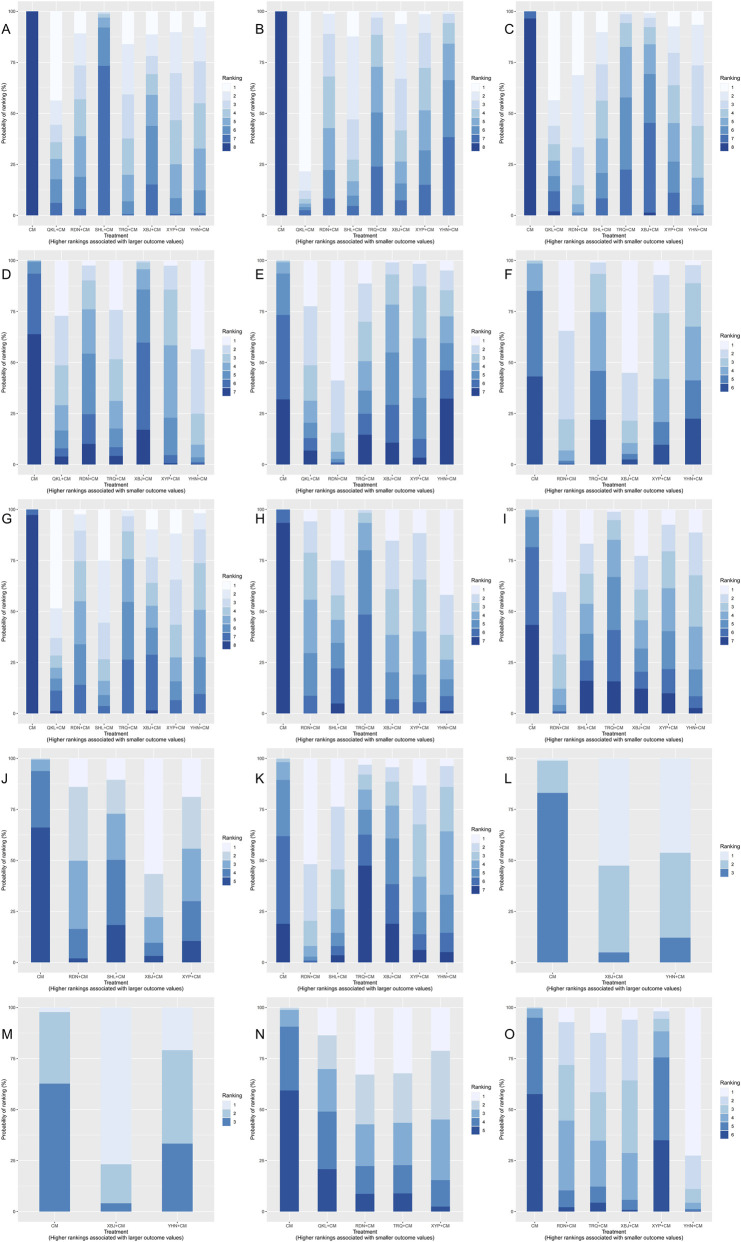
Rankogram plot. **(A)** Total clinical effective rate; **(B)** Antipyretic time; **(C)** Cough disappearance time; **(D)** TNF-α level; **(E)** IL-6 level; **(F)** Incidence of adverse reactions; **(G)** Disappearance time of lung rales; **(H)** Duration of asthma; **(I)** Hospitalization time; **(J)** CD4 level; **(K)** CD4/CD8 ratio; **(L)** IgM level; **(M)** IgG level; **(N)** IL-8 level; **(O)** hs-CRP level.

**FIGURE 6:**
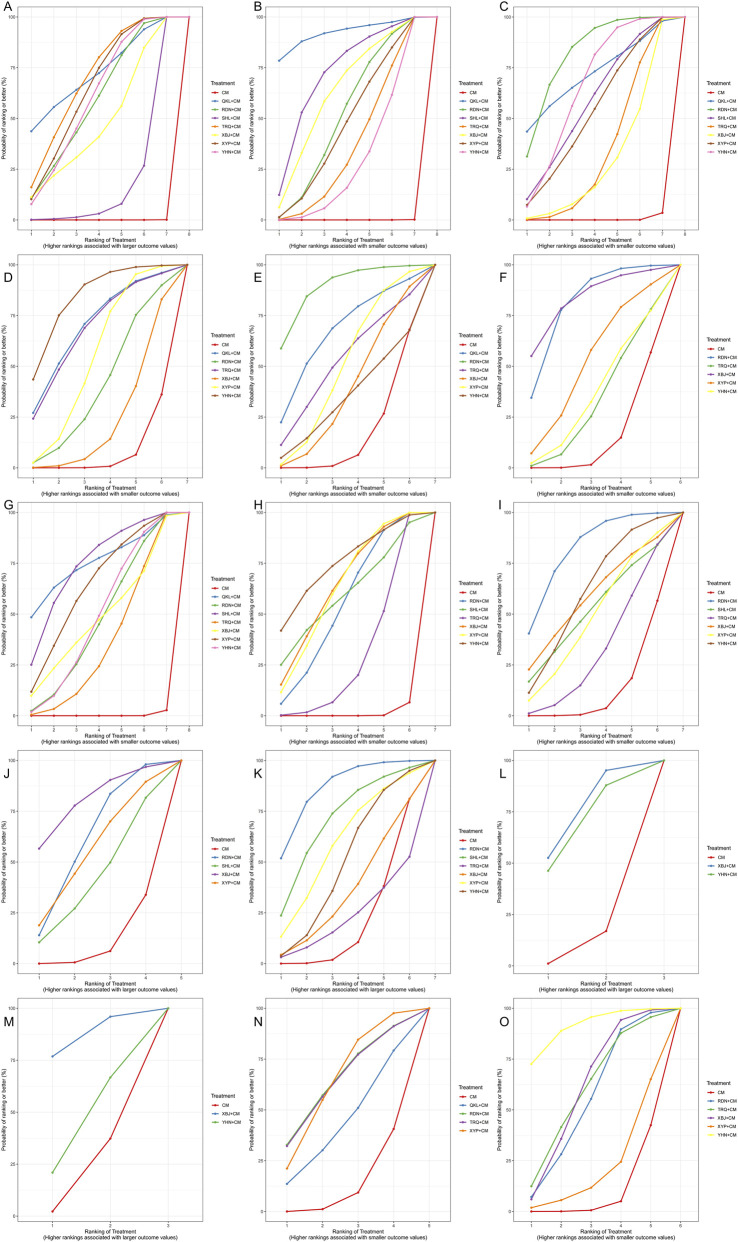
SUCRA plot. **(A)** Total clinical effective rate; **(B)** Antipyretic time; **(C)** Cough disappearance time; **(D)** TNF-α level; **(E)** IL-6 level; **(F)** Incidence of adverse reactions; **(G)** Disappearance time of lung rales; **(H)** Duration of asthma; **(I)** Hospitalization time; **(J)** CD4 level; **(K)** CD4/CD8 ratio; **(L)** IgM level; **(M)** IgG level; **(N)** IL-8 level; **(O)** hs-CRP level.

**FIGURE 7:**
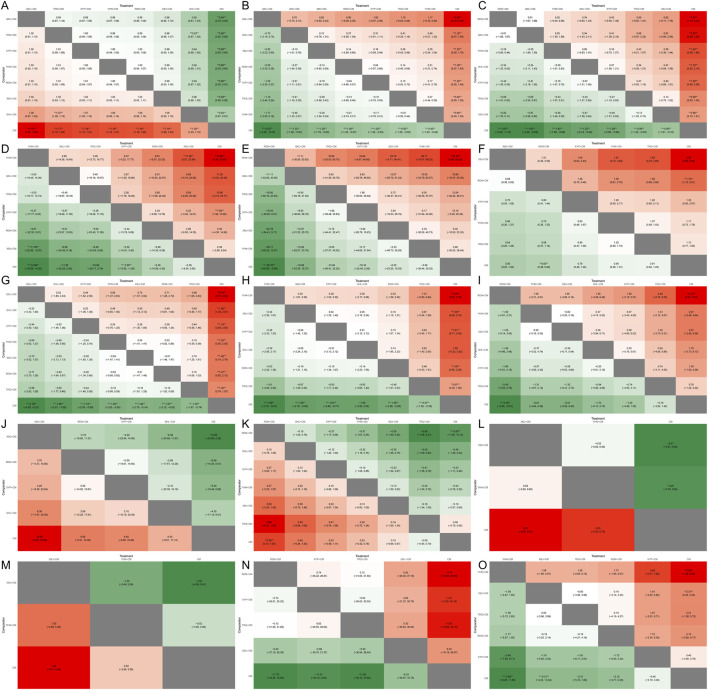
Heat map. **(A)** Total clinical effective rate; **(B)** Antipyretic time; **(C)** Cough disappearance time; **(D)** TNF-α level; **(E)** IL-6 level; **(F)** Incidence of adverse reactions; **(G)** Disappearance time of lung rales; **(H)** Duration of asthma; **(I)** Hospitalization time; **(J)** CD4 level; **(K)** CD4/CD8 ratio; **(L)** IgM level; **(M)** IgG level; **(N)** IL-8 level; **(O)** hs-CRP level.

A total of sixty-two studies (Qin and Wang, 2003; Hu and Zhu, 2006; Yang, 2007; Wang et al., 2008; Li, 2010; Liu et al., 2010a; Liu et al., 2010b; Wang, 2010; Li, 2011; Xiang et al., 2011; Cao and Xue, 2012; Cheng, 2012; Gao, 2012; Liu et al., 2012; Wang and Jin, 2012; Yang, 2012; Jiang, 2013; Li and Wu, 2013; Li, 2013; Qing, 2013; Shi and Yan, 2013; Zhao, 2013; Kang, 2015; Zhang and Zhou, 2015; Luo, 2016; Wang et al., 2016; Wu et al., 2016; Xiao, 2016; Yin et al., 2016; Zhao et al., 2016; Chen, 2017; Guo, 2017; Li et al., 2017; Li, 2017; Liu et al., 2017; Zeng, 2018; Gao C. et al., 2018; Liang, 2018; Liu, 2018; Yu, 2018; Zhang, 2018; Zhang et al., 2018; Zhao et al., 2018; Wang and Liu, 2019; Zhang, 2019; Fan et al., 2020; Lan and Yan, 2020; Li and Zhang, 2020; Lin et al., 2020; Qiao and He, 2020; Zhang et al., 2020; Li and Xiong, 2021; Xu, 2021; Zuo, 2021; Luo et al., 2022; Mo et al., 2022; Chang, 2023; Li, 2024; Wang et al., 2024; Mao et al., 2024; Cai, 2025; Yang et al., 2025) involving the seven types of TCMIs reported the total clinical efficacy rate (the total clinical effective rate is calculated as the difference between the total number of cases participating in the treatment and the number of treatment-ineffective cases as a percentage of the total number of cases participating in the treatment) involving the seven kinds of TCMIs. The forest plot (Figure 4A) demonstrated that seven kinds of TCMIs, when combined with CM, were significantly superior to CM alone (*P* < 0.05). The SUCRA and rankogram plots (Figures 5A, 6A) revealed the following ranking: QKL + CM (73.13%), TRQ + CM (70.25%), XYP + CM (65.65%), YHN + CM (61.61%), RDN + CM (59.97%), XBJ + CM (49.42%), SHL + CM (19.96%), and CM (0.01%). As presented in the heat map (Figure 7A), the QKL + CM (RR = 1.19, 95% CI: 1.07, 1.33) achieved the highest total effective rate, whereas SHL + CM (RR = 1.09, 95% CI: 1.03, 1.15) had a relatively lower rate.

#### 4.1.2 Antipyretic time

A total of fifty-four studies (Zou and Wang, 2000; Qin and Wang, 2003; Yang, 2007; Huang, 2008; Wang et al., 2008; Guo, 2009; Liu et al., 2010b; Xiang et al., 2011; Ye and Su, 2011; Cao and Xue, 2012; Cheng, 2012; Liu et al., 2012; Wang and Jin, 2012; Jiang, 2013; Li and Wu, 2013; Li, 2013; Qing, 2013; Shi and Yan, 2013; Zhao, 2013; Lin, 2015; Xue, 2015; Wang et al., 2016; Wu et al., 2016; Yin et al., 2016; Zhao et al., 2016; Chen, 2017; Li et al., 2017; Luo, 2017; Zeng, 2018; Liang, 2018; Liu, 2018; Zhang, 2018; Zhang, 2019; Jiang and Zuo, 2020; Lan and Yan, 2020; Li and Pan, 2020; Li and Zhang, 2020; Lin et al., 2020; Qiao and He, 2020; Li and Xiong, 2021; Su, 2021; Xu, 2021; Zhang et al., 2021; Gao et al., 2022; Luo et al., 2022; Mo et al., 2022; Chang, 2023; Gao and Du, 2023; Li, 2024; Liu, 2024; Wang et al., 2024; Mao et al., 2024; Cai, 2025; Yang et al., 2025) reported the antipyretic time of the seven kinds of TCMIs. The forest plot (Figure 4B) indicated that the seven types of TCMIs, when combined with CM, demonstrated superior efficacy compared to CM alone (*P* < 0.05). The SUCRA and rankogram plots (Figures 5B, 6B) ranked the treatments as follows: QKL + CM (92.28%), SHL + CM (72.44%), XBJ + CM (64.05%), RDN + CM (53.01%), XYP + CM (48.76%), TRQ + CM (38.25%), YHN + CM (28.84%), and CM (0.02%). As shown in the heat map (Figure 7B), QKL + CM (MD = −2.02, 95% CI: −3.30, −0.74) demonstrated the best antipyretic effect among those evaluated.

#### 4.1.3 Cough disappearance time

**FIGURE 8:**
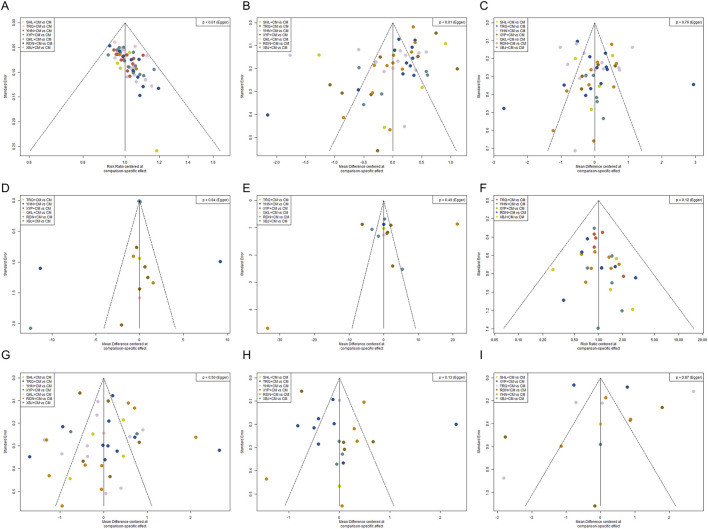
Funnel plot. **(A)** Total clinical effective rate; **(B)** Antipyretic time; **(C)** Cough disappearance time; **(D)** TNF-α level; **(E)** IL-6 level; **(F)** Incidence of adverse reactions; **(G)** Disappearance time of lung rales; **(H)** Duration of asthma; **(I)** Hospitalization time.

A total of forty-eight studies (Zou and Wang, 2000; Qin and Wang, 2003; Yang, 2007; Huang, 2008; Wang et al., 2008; Liu et al., 2010b; Ye and Su, 2011; Cheng, 2012; Liu et al., 2012; Jiang, 2013; Li, 2013; Qing, 2013; Shi and Yan, 2013; Zhao, 2013; Lin, 2015; Xue, 2015; Luo, 2016; Wang et al., 2016; Wu et al., 2016; Yin et al., 2016; Zhao et al., 2016; Chen, 2017; Li et al., 2017; Zeng, 2018; Liang, 2018; Liu, 2018; Zhang, 2018; Zhang et al., 2018; Zhang, 2019; Jiang and Zuo, 2020; Lan and Yan, 2020; Li and Pan, 2020; Li and Zhang, 2020; Lin et al., 2020; Qiao and He, 2020; Li and Xiong, 2021; Su, 2021; Xu, 2021; Gao et al., 2022; Luo et al., 2022; Chang, 2023; Gao and Du, 2023; Li, 2024; Liu, 2024; Wang et al., 2024; Mao et al., 2024; Cai, 2025; Yang et al., 2025) reported the cough disappearance time, involving the seven kinds of TCMIs. The forest plot (Figure 4C) showed that the seven TCMIs combined with CM had significant advantages over CM in terms of reducing cough time (*P* < 0.05). The SUCRA and rankogram plots (Figures 5C, 6C) indicated the following ranking: RDN + CM (82.31%), QKL + CM (72.13%), YHN + CM (66.38%), SHL + CM (59%), XYP + CM (54.45%), TRQ + CM (34.95%), XBJ + CM (30.27%), and CM (0.51%). Figure 7C provided an overview of the heat map, revealing that the RDN + CM (MD = −1.67, 95% CI: −2.23, −1.13) had the shortest cough disappearance time. The comparison-corrected funnel plot (Figure 8C) showed *P* > 0.05, indicating no publication bias.

#### 4.1.4 TNF-α level

Sixteen studies (Wu et al., 2016; Wang et al., 2016; Li et al., 2017; Gao C. et al., 2018; Song and Yang, 2018; Zhao et al., 2018; Jing, 2018; Qiao and He, 2020; Li and Zhang, 2020; Lin et al., 2020; Su, 2021; Luo et al., 2022; Mo et al., 2022; Gao et al., 2022; Mao et al., 2024; Cai, 2025) reported TNF-α level, involving six TCMIs. The forest plot (Figure 4D) indicated that both YHN + CM (MD = −13.96, 95% CI: −23.65, −4.35) and XYP + CM (MD = −7.69, 95% CI: −13.92, −1.50) were significantly superior to CM (*P* < 0.05). Based on the SUCRA and rankogram plots (Figures 5D, 6D), the treatments were ranked as follows: YHN + CM (84.01%), QKL + CM (70.14%), TRQ + CM (68.55%), XYP + CM (55.04%), RDN + CM (41.2%), XBJ + CM (23.82%), CM (7.24%). The results from the heat map are presented in Figure 7D. Finally, the comparison-corrected funnel plot (Figure 8D) showed no significant asymmetry (*P* > 0.05), indicating a low likelihood of publication bias.

#### 4.1.5 IL-6 level

Fourteen studies (Wang et al., 2016; Wu et al., 2016; Li et al., 2017; Song and Yang, 2018; Gao C. et al., 2018; Jing, 2018; Qiao and He, 2020; Qin et al., 2020; Lin et al., 2020; Su, 2021; Mo et al., 2022; Gao and Du, 2023; Wang et al., 2024; Cai, 2025) reported the IL-6 level after six TCMIs. The forest plot (Figure 4E) indicated that RDN + CM (MD = −30.10, 95% CI: −55.29, −5.98) was superior to CM (*P* < 0.05). As shown in Figures 5E, 6E, the ranking of treatment efficacy was as follows: RDN + CM (88.8%), QKL + CM (67.06%), TRQ + CM (52.51%), XYP + CM (50.67%), XBJ + CM (39.1%), YHN + CM (34.83%), and CM (17.02%). The results are further visualized in the heat map (Figure 7E). Additionally, the comparison correction funnel plot (Figure 8E) revealed no evidence of publication bias (*P* > 0.05).

#### 4.1.6 Incidence of adverse reactions

The incidence of adverse reactions was reported in thirty studies (Liu et al., 2012; Xue, 2015; Lin, 2015; Luo, 2016; Wang et al., 2016; Zhao et al., 2016; Luo, 2017; Liu et al., 2017; Zhang et al., 2018; Liu, 2018; Liang, 2018; Gao C. et al., 2018; Zeng, 2018; Lin et al., 2020; Zhang et al., 2020; Li and Zhang, 2020; Fan et al., 2020; Zuo, 2021; Hu et al., 2021; Luo et al., 2022; Mo et al., 2022; Gao et al., 2022; Chang, 2023; Gao and Du, 2023; Liu, 2024; Mao et al., 2024; Li, 2024; Wang et al., 2024; Cai, 2025; Yang et al., 2025) recorded eight cases of nausea and vomiting, nine cases of abdominal pain and diarrhea, six cases of rash, three cases of leukopenia, and four cases of liver function damage after RDN + CM treatment. According to the SUCRA and rankogram plots (Figures 5F, 6F), the treatments were ranked as follows: XBJ + CM (83.07%), RDN + CM (80.67%), XYP + CM (52.11%), YHN + CM (36.44%), TRQ + CM (33.03%), and CM (14.67%). Although RDN + CM ranked second (RR = 0.60, 95% CI: 0.38, 0.89), the forest plot (Figure 4F) revealed its significant advantage in drug safety (*P* < 0.05). The heat map is shown in Figure 7F. Moreover, the comparison-corrected funnel plot (Figure 8F) showed *P* > 0.05, suggesting no publication bias.

Among the thirty studies, eight studies (Lin, 2015; Zhao et al., 2016; Luo, 2017; Zeng, 2018; Zuo, 2021; Luo et al., 2022; Gao and Du, 2023; Li, 2024) recorded eight cases of nausea and vomiting, nine cases of abdominal pain and diarrhea, six cases of rash, three cases of leukopenia, and four cases of liver function damage after RDN + CM treatment. Six studies (Liu et al., 2012; Lin et al., 2020; Fan et al., 2020; Hu et al., 2021; Chang, 2023; Mao et al., 2024) recorded seven cases of allergic reactions, five cases of pyrogen-like reactions, thirteen cases of gastrointestinal reactions, three cases of dizziness and headache, and three cases of liver function damage after YHN + CM treatment. Seven studies (Wang et al., 2016; Liu et al., 2017; Gao C. et al., 2018; Zhang et al., 2018; Mo et al., 2022; Liu, 2024; Cai, 2025) recorded one case of palpitations, thirteen cases of digestive tract reactions, four cases of allergic reactions, three case of dizziness and headache, two cases of irritability, one case of hemolytic anemia, two cases of decreased skin oxygenation, and two cases of drug-induced fever after XYP + CM treatment. Four studies (Zhang et al., 2020; Li and Zhang, 2020; Gao et al., 2022; Wang et al., 2024) recorded five cases of nausea and vomiting, one case of abdominal pain and diarrhea, one case of rash, one case of dizziness and headache, one case of dry cough, and one case of bleeding after XBJ + CM treatment. Five studies (Xue, 2015; Luo, 2016; Liang, 2018; Liu, 2018; Yang et al., 2025) recorded twenty cases of digestive tract reactions, thirteen cases of dizziness and headache, seven cases of rash, five cases of leukopenia, and two cases of allergic reactions after TRQ + CM treatment.

### 4.2 Secondary outcomes

#### 4.2.1 Disappearance time of lung rales

A total of forty-four studies (Zou and Wang, 2000; Qin and Wang, 2003; Yang, 2007; Huang, 2008; Wang et al., 2008; Guo, 2009; Liu et al., 2010b; Xiang et al., 2011; Ye and Su, 2011; Cheng, 2012; Liu et al., 2012; Wang and Jin, 2012; Jiang, 2013; Li, 2013; Qing, 2013; Shi and Yan, 2013; Zhao, 2013; Lin, 2015; Xue, 2015; Luo, 2016; Wang et al., 2016; Zhao et al., 2016; Chen, 2017; Li et al., 2017; Zeng, 2018; Liang, 2018; Liu, 2018; Zhang, 2018; Zhang, 2019; Jiang and Zuo, 2020; Lan and Yan, 2020; Li and Pan, 2020; Li and Zhang, 2020; Lin et al., 2020; Qiao and He, 2020; Li and Xiong, 2021; Xu, 2021; Gao et al., 2022; Luo et al., 2022; Mo et al., 2022; Chang, 2023; Li, 2024; Cai, 2025; Yang et al., 2025) reported the disappearance time of pulmonary rales, involving the seven kinds of TCMIs. The forest plot (Figure 4G) indicated that the seven types of TCMIs combined with CM were superior to CM alone (*P* < 0.05). The SUCRA and rankogram plots (Figures 5G, 6G) showed the following hierarchy of efficacy: QKL + CM (75.9%), SHL + CM (75.07%), XYP + CM (64.72%), YHN + CM (50.01%), XBJ + CM (49.17%), RDN + CM (47.88%), TRQ + CM (36.84%), and CM (0.39%). Figure 7G presents the heat map, with QKL + CM (MD = −2.18, 95% CI: −4.05, −0.31) showing the shortest disappearance time of pulmonary rales. Furthermore, the funnel plot (Figure 8G) of the comparison correction showed *P* > 0.05, and no publication bias was observed.

#### 4.2.2 Duration of asthma

Twenty-two studies (Qin and Wang, 2003; Huang, 2008; Guo, 2009; Wang and Jin, 2012; Lin, 2015; Xue, 2015; Luo, 2016; Wu et al., 2016; Yin et al., 2016; Zeng, 2018; Liang, 2018; Liu, 2018; Zhang, 2018; Zhang et al., 2018; Zhang, 2019; Lan and Yan, 2020; Su, 2021; Gao et al., 2022; Mo et al., 2022; Gao and Du, 2023; Li, 2024; Yang et al., 2025) reported the duration of asthma with six types of TCMIs. As shown in the forest plot (Figure 4H), there was no significant difference in efficacy between SHL + CM and CM (*P* > 0.05). The SUCRA and rankogram plots (Figures 5H, 6H) indicated the following rank order of efficacy: YHN + CM (75.11%), XBJ + CM (64.77%), XYP + CM (63.52%), SHL + CM (59.98%), RDN + CM (55.51%), TRQ + CM (29.98%), and CM (1.13%). The heat map in Figure 7H showed that YHN + CM (MD = −1.99, 95% CI: −3.70, −0.27) required the shortest time to relieve asthma (*P <* 0.05). Finally, the assessment for publication bias using a funnel plot (Figure 8H) showed no evidence of bias (*P* > 0.05).

#### 4.2.3 Hospitalization time

Fifteen studies (Zou and Wang, 2000; Wang et al., 2008; Huang, 2008; Guo, 2009; Ye and Su, 2011; Luo, 2016; Luo, 2017; Liu, 2018; Qin et al., 2020; Qiao and He, 2020; Luo et al., 2022; Zhang et al., 2023; Chang, 2023; Wang et al., 2024; Cai, 2025) involving six TCMIs reported the hospitalization time. In the forest plot (Figure 4I), RDN + CM (MD = −3.16, 95% CI: −5.43, −0.91) had an advantage over CM (*P* < 0.05). The SUCR and rankogram plots (Figures 5I, 6I) indicated the following efficacy ranking: RDN + CM (82.33%), YHN + CM (61.41%), XBJ + CM (58.67%), SHL + CM (52.27%), XYP + CM (49.13%), TRQ + CM (32.96%), and CM (13.21%). Figure 7I displayed the heat map. Furthermore, the comparison-corrected funnel plot (Figure 8I) was not significant (*P* > 0.05), suggesting the absence of publication bias.

#### 4.2.4 CD4 level

Six studies (Qing, 2013; Lin, 2015; Zhao et al., 2016; Zhai, 2018; Gao et al., 2022; Mo et al., 2022) reported the CD4 level after four TCMIs. Based on the SUCRA and rankogram plots (Figures 5J, 6J), the treatments ranked in the following order: XBJ + CM (80.41%), RDN + CM (61.46%), XYP + CM (55.67%), SHL + CM (42.27%), and CM (10.18%). A heat map further depicting these results is provided in Figure 7J.

#### 4.2.5 CD4/CD8 ratio

Nine studies (Qing, 2013; Lin, 2015; Zhai, 2018; Qin et al., 2020; Fan et al., 2020; Hu et al., 2021; Mo et al., 2022; Gao et al., 2022; Guo and Huang, 2023) reported the CD4/CD8 ratio involving six TCMIs. In the forest plot (Figure 4J), RDN + CM (MD = 0.60, 95% CI: 0.13, 1.05) demonstrated a significant advantage over CM (*P* < 0.05). According to the SUCRA and rankogram plots (Figures 5K, 6K), the treatments were ranked as follows: RDN + CM (86.61%), SHL + CM (71.02%), XYP + CM (59.84%), YHN + CM (50.13%), XBJ + CM (36.81%), TRQ + CM (23.61%), and CM (21.97%). This ranking is further demonstrated in the heat map (Figure 7K).

#### 4.2.6 IgM level

Three studies (Li and Zhang, 2020; Su, 2021; Chang, 2023) reported the IgM level after two TCMIs. The SUCRA and rankogram plots (Figures 5L, 6L) indicated that XBJ + CM (73.88%) had the highest probability of being the best treatment, followed by YHN + CM (67.1%), and then CM (9.03%). The corresponding heat map is shown in Figure 7L.

#### 4.2.7 IgG level

Three studies (Li and Zhang, 2020; Su, 2021; Chang, 2023) reported the IgG level after two TCMIs. The SUCRA and rankogram plots (Figures 5M, 6M) showed that XBJ + CM had the highest probability of being the best treatment (86.4%), followed by YHN + CM (43.83%), and CM (19.77%). A heat map graphically representing these comparison results is displayed in Figure 7M.

#### 4.2.8 IL-8 level

Six studies (Wang et al., 2016; Li et al., 2017; Song and Yang, 2018; Zhao et al., 2018; Qiao and He, 2020; Mo et al., 2022) reported IL-8 level after four TCMIs. The SUCRA and rankogram plots (Figures 5N, 6N) indicated the following ranking: RDN + CM (64.79%), XYP + CM (64.58%), TRQ + CM (64.29%), QKL + CM (43.52%), and CM (12.81%). The corresponding heat map is presented in Figure 7N.

#### 4.2.9 hs-CRP level

Nine studies (Wu et al., 2016; Zhao et al., 2016; Jing, 2018; Qiao and He, 2020; Hu et al., 2021; Su, 2021; Gao and Du, 2023; Chang, 2023; Cai, 2025) reported the hs-CRP level after four TCMIs. The forest plot (Figure 4K) demonstrated the superiority of both YHN + CM (MD = −3.89, 95% CI: −6.29, −1.38) and XBJ + CM (MD = −2.31, 95% CI: −4.32, −0.34) over CM (*P* < 0.05). Based on the SUCRA and rankogram plots (Figures 5O, 6O), the ranking was as follows: YHN + CM (91.14%), XBJ + CM (61.31%), TRQ + CM (60.53%), RDN + CM (55.66%), XYP + CM (21.74%), CM (9.63%). The heat map illustrating these findings is provided in Figure 7O.

### 4.3 Sensitivity analysis

Through sensitivity analysis, we evaluated the primary outcomes and secondary outcomes. The results show that when each individual study was excluded one by one, no significant changes were observed in most outcomes (Supplementary Material S4), which confirms the reliability of the research findings. There are cases where the incidence of adverse reactions and IgM level exceed the invalid line, indicating a lack of robustness. Therefore, caution should be exercised when dealing with these two outcomes.

### 4.4 Subgroup analysis

Age-based subgroup analyses were conducted for all eligible studies, whereas treatment-duration subgroup analyses were feasible only for a limited number of comparisons because treatment-duration information was insufficiently reported in most studies. The results showed that QKL + CM achieved a higher overall clinical response rate in children than in adults, whereas XBJ + CM was more effective in adults than in children. Regarding the disappearance time of lung rales, QKL + CM was more effective in children than in adults, while RDN + CM was associated with shorter hospital stays in adults compared to children. Furthermore, we observed that both RDN + CM and TRQ + CM demonstrated favorable performance in reducing fever and alleviating cough in both adults and children, suggesting their potential for broad applicability. In contrast, XBJ + CM showed notable efficacy primarily in adults. Regarding the modulation of inflammatory factors, QKL + CM and XYP + CM were more effective in reducing TNF-α levels in children than in adults, while RDN + CM showed favorable efficacy in lowering IL-6 levels in adults. Additionally, during short-term treatment (<7 days), XYP + CM and RDN + CM exhibited significant inhibitory effects on TNF-α and IL-6 levels, respectively. With extended treatment duration, QKL + CM, TRQ + CM, XYP + CM, and YHN + CM demonstrated more pronounced effects in reducing TNF-α level (Supplementary Material S4). These findings suggest that different therapeutic regimens may exhibit varying efficacy across distinct populations and treatment durations. However, due to limitations in the quantity and quality of available studies, further high-quality research is needed for validation.

### 4.5 Funnel plot features

The results for cough disappearance time, TNF-α level, IL-6 level, incidence of adverse reactions, disappearance time of lung rales, duration of asthma, and hospitalization time were generally symmetrical, suggesting an absence of publication bias. In contrast, other studies exhibited publication bias. More details can be found in Figure 8.

## 5 Discussion

In this study, 83 RCTs were combined to evaluate the efficacy and safety of seven types of heat-clearing and detoxifying TCMIs for viral pneumonia using NMA. The results showed that the TCMIs combined with CM have generally produced satisfactory outcomes. In terms of efficacy, RDN + CM was the most effective treatment for shortening cough disappearance and hospitalization time, lowering IL-6 level, and increasing the CD4/CD8 ratio. QKL + CM had the greatest effect on increasing the total clinical effective rate, shortening the antipyretic time, and decreasing the disappearance time of lung rales. YHN + CM showed significant benefits in decreasing the duration of asthma and reducing TNF-α and hs-CRP levels. However, TCMIs + CM were similarly successful as CM in reducing IL-8, CD4, IgM, and IgG levels. Furthermore, RDN + CM did not result in a higher incidence of adverse safety events compared with the control group, suggesting that RDN + CM may represent a safe therapeutic approach.

In recent years, a number of studies have utilized NMA to systematically evaluate the efficacy of TCM in treating viral pneumonia. For example, Zhu et al. focused on the application of TRQ (Zhu et al., 2020). Later, Li et al. broadened the scope to include a wider variety of TCMIs (Li et al., 2022). Their study emphasized clinical symptom improvement and concluded that the combination of RDN with conventional antiviral drugs produced the best outcomes in terms of shortening hospital stays and reducing adverse events, which is consistent with our own results. Compared with these earlier studies, the present research incorporates a substantial number of recent RCTs. It not only examines clinical symptom improvement but also integrates inflammatory cytokine levels and immune function indicators into the outcome assessment, thereby evaluating the efficacy of TCMIs from a more comprehensive perspective. Furthermore, through subgroup analyses, we explore potential differences in the efficacy of various heat-clearing and detoxifying TCMIs based on patient age and duration of intervention, thereby enriching the evidence base in this field.

Viral pneumonia is a common respiratory disease that belongs to the categories of “wind-warm lung heat,” “seasonal cold,” and “epidemic disease” in TCM. Under physiological conditions, the lung immune cells are relatively resting. During viral infection, activation of the immune cells in the lungs induces the production of pro-inflammatory cytokines. Simultaneously, inflammatory mediators recruit a large number of immune cells, triggering an inflammatory response. Inflammatory factors induce immune cell activation and form an excessive inflammatory response, leading to cytokine storms, which can cause extensive lung injury and respiratory distress syndrome. Modern medicine mainly adopts symptomatic and supportive treatments combined with antiviral drugs, glucocorticoids, human immunoglobulins, and other drugs. However, due to the variability of the virus, the lack of specific pathogen treatment methods has brought great challenges to clinical treatment. At the same time, there are some limitations to CM treatment alone, such as a high incidence of adverse reactions, long duration of symptom relief, and limited ability to reduce the level of inflammatory factors (Chun et al., 2021). In addition, CM has restrictions on children’s medication, as well as a lag in research and development. CM combined with TCMIs can effectively compensate for these shortcomings. TCMIs have advantages in improving clinical efficacy, relieving respiratory symptoms, reducing inflammatory factor levels, and minimizing the occurrence of adverse events. As the pathogenesis of viral pneumonia often involves lung qi stagnation and the invasion of pathogenic heat, TCMIs with heat-clearing and detoxifying properties are widely applied in clinical management. RDN, QKL, and YHN are common TCMIs used to clear heat and to detoxify. RDN is primarily composed of Artemisia annua, honeysuckle, and gardenia groups. Research has shown its effectiveness in reducing mortality, pulmonary edema, and cough frequency in mice infected with the influenza virus. Additionally, RDN could reduce the white blood cell count and downregulate the levels of IL-6 and IL-10 (Ma et al., 2016; Zhang et al., 2019). In acute lung injury rabbit models, RDN reduced the levels of IL-8 and TNF-α in plasma and bronchoalveolar lavage fluid, which also demonstrated strong neuraminidase inhibitory activity *in vitro* (Liu et al., 2007). QKL consists of Radix Isatidis, cholic acid, baicalin, honeysuckle, buffalo horn, mother-of-pearl, gardenia, hyodeoxycholic acid, and other metabolites. QKL has been found to reduce TNF-α levels while increasing the levels of nitric oxide and superoxide dismutase to alleviate lung injury in rats. Further experiments revealed that QKL increased the richness of beneficial microbial flora and inhibited inflammatory metabolic pathways, such as those involving arachidonic acid and glycerophospholipid metabolism, thereby alleviating pneumonia in rats (Chen et al., 2023; Gao X. et al., 2018). Finally, YHN, which consists of andrographolide and other metabolites, has been shown to reduce TNF-α and IL-10 levels in mice with viral pneumonia (Luo et al., 2014). In addition, existing studies suggested that adverse events, such as allergic reactions, may occur after the application of TCMIs, but most patients experience alleviated adverse reactions after intervention or treatment (Huang et al., 2021). While modern pharmaceutical technology has improved the quality of TCMIs, standardized clinical research data and real-world evidence still need to be supplemented, and the mechanisms and risk factors of adverse reactions need to be studied further (Zheng et al., 2022).

Although several heat-clearing and detoxifying TCMIs used in conjunction with CM appeared to show favorable effects in patients with viral pneumonia, these findings should be interpreted cautiously. The evidence network was largely star-shaped, with most interventions being compared only with CM, and direct evidence between different TCMIs being scarce. Under such circumstances, differences across TCMIs were inferred mainly through indirect comparisons, which may reduce confidence in the stability of the comparative results. This concern is further amplified by variations across the included trials in terms of study quality, treatment regimens, and clinical characteristics. In addition, SUCRA rankings are useful for describing the relative ordering of interventions within the network, but they should not be equated with certainty of evidence or taken as a basis for firm clinical preference. This concern is reinforced by the GRADE evaluation, which indicates that the certainty of evidence for most comparisons was not high. Therefore, the observed ranking patterns are better understood as signals that may guide future research priorities rather than as definitive evidence for decision-making in clinical practice.

Nevertheless, this study still has important clinical and research implications. On the one hand, by systematically synthesizing the currently available published evidence, this study provides a relatively comprehensive overview of the use of heat-clearing and detoxifying TCMIs in the treatment of viral pneumonia, thereby helping to reduce bias arising from overreliance on individual positive studies or selective citation of the literature. On the other hand, our findings also suggest that, despite their widespread clinical use, the overall evidence base supporting these interventions remains relatively weak, and substantial improvements are still needed in study design, methodological quality, and reporting standards. It is noteworthy that although no definitive conclusions can be drawn because of the limitations in the quality of the original studies, several TCMIs demonstrated relatively consistent trends toward benefit across major outcomes. This signal may provide a basis for generating hypotheses for future research. High-quality RCTs with rigorous design, adequate sample size, standardized outcome measures, and direct comparative evidence are still required to further verify the true efficacy and clinical value of different heat-clearing and detoxifying TCMIs in the treatment of viral pneumonia.

## 6 Limitations

This study had some limitations. First, the quality of the included studies was generally low, with only a few studies employing randomization and blinding methods. Additionally, some outcome measures, such as assessment of the total clinical efficacy rate, were subjective and may introduce publication bias. Furthermore, although most of the included studies did not report outcomes disaggregated by sex, we acknowledge that sex is a critical biological variable. We therefore recognize this as a limitation and emphasize the need for future studies to explore the potential sex-specific effects of TCMIs in the treatment of viral pneumonia. Moreover, limitations in confounding control should be noted, underscoring the importance of conducting future stratified analyses or analyses based on individual patient data. Finally, the study of adverse reactions remains to be elucidated through larger-scale reports in the future, and this analysis included only 83 RCTs, highlighting the need for a larger number of clinical trials to verify our conclusions.

## 7 Conclusion

This study showed that heat-clearing and detoxifying TCMIs combined with CM are more beneficial than CM alone for the treatment of viral pneumonia. A comprehensive comparison showed that RDN is a relatively good intervention measure. However, owing to the limitations of this study, large-sample and high-quality RCTs are needed to supplement and improve the results.

## Data Availability

The original contributions presented in the study are included in the article/Supplementary Material, further inquiries can be directed to the corresponding author.
