# Supplementary material for: Therapeutic effects of traditional Chinese medicine injections with heat-clearing and detoxifying properties on viral pneumonia: a systematic review and network meta-analysis
Source: Front Pharmacol. 2026 May 14;17:1771777. doi: 10.3389/fphar.2026.1771777 (PMC13216718; doi:10.3389/fphar.2026.1771777)
Supplement: Supplementary file 1 [file Supplementaryfile6.docx]

**Supplementary Material 6: Certainty of the evidence**

| **Certainty assessment** | | | | | | | **№ of patients** | | **Effect** | | **Certainty** | **Importance** |
| --- | --- | --- | --- | --- | --- | --- | --- | --- | --- | --- | --- | --- |
| **№ of studies** | **Study design** | **Risk of bias** | **Inconsistency** | **Indirectness** | **Imprecision** | **Other considerations** | **Treatment** | **Control** | **Relative (95% CI)** | **Absolute (95% CI)** |  |  |
| **Total clinical effective rate** | | | | | | | | | | | | |
| 62 | randomised trials | not serious | not serious | not serious | not serious | publication bias strongly suspected^a^ | 3213/3413 (94.1%) | 2657/3328 (79.8%) | **RR 1.1796** (1.1575 to 1.2021) | **143 more per 1,000** (from 126 more to 161 more) | ⨁⨁⨁◯ Moderate^a^ | CRITICAL |
| **Antipyretic time** | | | | | | | | | | | | |
| 54 | randomised trials | not serious | not serious | not serious | not serious | publication bias strongly suspected^a^ | 2622 | 2559 | - | **1.0166 fewer** (1.1856 fewer to 0.8475 fewer) | ⨁⨁⨁◯ Moderate^a^ | CRITICAL |
| **Cough disappearance time** | | | | | | | | | | | | |
| 48 | randomised trials | not serious | not serious | not serious | not serious | none | 2356 | 2298 | - | **1.2839 fewer** (1.5053 fewer to 1.0626 fewer) | ⨁⨁⨁⨁ High | CRITICAL |
| **TNF-α level** | | | | | | | | | | | | |
| 16 | randomised trials | not serious | not serious | not serious | not serious | none | 914 | 912 | - | **6.9331 fewer** (10.0065 fewer to 3.8597 fewer) | ⨁⨁⨁⨁ High | CRITICAL |
| **IL-6 level** | | | | | | | | | | | | |
| 14 | randomised trials | not serious | not serious | not serious | not serious | none | 762 | 767 | - | **12.0843 fewer** (18.8935 fewer to 5.275 fewer) | ⨁⨁⨁⨁ High | CRITICAL |
| **Incidence of adverse reactions** | | | | | | | | | | | | |
| 30 | randomised trials | not serious | not serious | not serious | not serious | none | 144/1424 (10.1%) | 161/1409 (11.4%) | **RR 0.8712** (0.7072 to 1.0732) | **15 fewer per 1,000** (from 33 fewer to 8 more) | ⨁⨁⨁⨁ High | CRITICAL |
| **Disappearance time of lung rales** | | | | | | | | | | | | |
| 44 | randomised trials | not serious | not serious | not serious | not serious | none | 2197 | 2136 | - | **1.5256 fewer** (1.7879 fewer to 1.2633 fewer) | ⨁⨁⨁⨁ High | IMPORTANT |
| **Duration of asthma** | | | | | | | | | | | | |
| 22 | randomised trials | serious^b^ | not serious | not serious | not serious | none | 1088 | 1078 | - | **1.3578 fewer** (1.6878 fewer to 1.0278 fewer) | ⨁⨁⨁◯ Moderate^b^ | IMPORTANT |
| **Hospitalization time** | | | | | | | | | | | | |
| 15 | randomised trials | not serious | not serious | not serious | not serious | none | 663 | 670 | - | **1.8759 fewer** (2.7561 fewer to 0.9958 fewer) | ⨁⨁⨁⨁ High | IMPORTANT |
| **CD4 level** | | | | | | | | | | | | |
| 6 | randomised trials | serious^b^ | not serious | not serious | not serious | publication bias strongly suspected^a^ | 259 | 253 | - | **7.039 more** (4.3825 more to 9.6955 more) | ⨁⨁◯◯ Low^a,b^ | IMPORTANT |
| **CD4/CD8 ratio** | | | | | | | | | | | | |
| 9 | randomised trials | serious^b^ | not serious | not serious | not serious | publication bias strongly suspected^a^ | 379 | 377 | - | **0.3506 more** (0.1747 more to 0.5264 more) | ⨁⨁◯◯ Low^a,b^ | IMPORTANT |
| **IgM level** | | | | | | | | | | | | |
| 3 | randomised trials | serious^b^ | not serious | not serious | not serious | publication bias strongly suspected^a^ | 158 | 157 | - | **0.2633 higher** (0.1304 higher to 0.3961 higher) | ⨁⨁◯◯ Low^a,b^ | IMPORTANT |
| **IgG level** | | | | | | | | | | | | |
| 3 | randomised trials | serious^b^ | not serious | not serious | not serious | publication bias strongly suspected^a^ | 158 | 157 | - | **1.3708 more** (0.1048 more to 2.6367 more) | ⨁⨁◯◯ Low^a,b^ | IMPORTANT |
| **IL-8 level** | | | | | | | | | | | | |
| 6 | randomised trials | not serious | not serious | not serious | not serious | publication bias strongly suspected^a^ | 339 | 338 | - | **10.4616 lower** (14.3411 lower to 6.582 lower) | ⨁⨁⨁◯ Moderate^a^ | IMPORTANT |
| **hs-CRP leve** | | | | | | | | | | | | |
| 9 | randomised trials | serious^b^ | not serious | not serious | not serious | publication bias strongly suspected^a^ | 536 | 534 | - | **2.4002 lower** (3.2607 lower to 1.5396 lower) | ⨁⨁◯◯ Low^a,b^ | IMPORTANT |

**CI:** confidence interval; **RR:** risk ratio

^a^Funnel plot analysis indicated a potential risk of publication bias.

^b^The study design suffered from major biases affecting randomization and the distribution of findings
