# Supplementary material for: Therapeutic effects of traditional Chinese medicine injections with heat-clearing and detoxifying properties on viral pneumonia: a systematic review and network meta-analysis
Source: Front Pharmacol. 2026 May 14;17:1771777. doi: 10.3389/fphar.2026.1771777 (PMC13216718; doi:10.3389/fphar.2026.1771777)
Supplement: Supplementary file 3 [file Supplementaryfile5.docx]

## Supplement Material 5: Detail information of the traditional Chinese medicine injections (TCMIs)

## 1 Composition of the TCMIs

| TCMIs | Source | Batch number | Constituent(s) | Usage and  dosage | Completeness of reporting in the original study |
| --- | --- | --- | --- | --- | --- |
| Reduning Injection | Jiangxi Kangyuan Pharmaceutical Co., Ltd. | Z20050217 | *Artemisia annua* L. [Asteraceae; Artemisiae Annuae Herba], *Gardenia jasminoides* J. Ellis [Rubiaceae; Gardeniae Fructus], *Lonicera japonica* Thunb. [Caprifoliaceae; Lonicerae Japonicae Flos] | Adults: 20mL, Qd, ivgtt  Children: 10-20mL, Qd, ivgtt | Inadequate |
| Xiyanping Injection | Jiangxi Qingfeng Pharmaceutical Co., Ltd. | Z20026249 | *Andrographis paniculata* (Burm.f.) Wall. ex Nees [Acanthaceae; Andrographis herba] | Adults: 250-500mg, Qd, ivgtt  Children: 5-10mg/kg, Qd, ivgtt | Inadequate |
| Yanhuning Injection | Hainan Lingkang Pharmaceutical Co., Ltd.  Jilin Mayinglong Pharmaceutical Co., Ltd.  Shandong Ruiyang Pharmaceutical Co., Ltd.  Hainan General Kone Pharmaceutical Co., Ltd.  Chongqing Yuyou Pharmaceutical Co., Ltd.  Beijing SL Pharmaceutical Co., Ltd. | H20046110; H20055351; H20068102; H20061057; H20065443; H50021641; H20061009 | *Andrographis paniculata* (Burm.f.) Wall. ex Nees [Acanthaceae; Andrographis herba] | 0.16-0.4g, Qd/Bid, ivgtt | Inadequate |
| Tanreqing Injection | Shanghai Kaibao Pharmaceutical Co., Ltd. | Z20030054 | *Scutellaria baicalensis* Georgi [Lamiaceae; Scutellariae radix], *Selenarctos thibetanus* G.Cuvier [Ursidae; Fel Selenarcti et Ursi], *Naemorhedus goral* Hardwicke [Bovidae; Cornu Oryx], *Lonicera japonica* Thunb. [Caprifoliaceae; Lonicerae japonicae flos], *Forsythia suspensa* (Thunb.) Vahl [Oleaceae; Forsythiae fructus] | Adults: 20-40mL, Qd, ivgtt  Children: 0.3-0.5mL/kg, Qd, ivgtt | Inadequate |
| Xuebijing Injection | Tianjin Hongri Pharmaceutical Co., Ltd. | Z20040033 | *Carthamus tinctorius* L. [Asteraceae; Carthami flos], *Paeonia lactiflora* Pall. [Paeoniaceae; Paeoniae radix rubra], Conioselinum anthriscoides 'Chuanxiong' [Apiaceae; Chuanxiong rhizoma], *Salvia miltiorrhiza* Bunge [Lamiaceae; Salviae miltiorrhizae radix et rhizoma], *Angelica sinensis* (Oliv.) Diels [Apiaceae; Angelicae sinensis radix] | 50-100mL, Bid, ivgtt | Inadequate |
| Shuanghuanglian Injection | Harbin Zhenbao Pharmaceutical Co., Ltd.  Heilongjiang Songhuajiang Pharmaceutical Co., Ltd.  Henan Fushen Pharmaceutical Co., Ltd. | Z10940044 | *Lonicera japonica* Thunb. [Caprifoliaceae; Lonicerae japonicae flos], *Forsythia suspensa* (Thunb.) Vahl [Oleaceae; Forsythiae fructus], *Scutellaria baicalensis* Georgi [Lamiaceae; Scutellariae radix], *Selenarctos thibetanus* G.Cuvier [Ursidae; Fel Selenarcti et Ursi] | 1mL·kg^-1^, Qd, ivgtt | Inadequate |
| Qingkailing Injection | Henan Shennong Pharmaceutical Co., Ltd. | 20140105 | *Scutellaria baicalensis* Georgi [Lamiaceae; Scutellariae radix], *Selenarctos thibetanus* G.Cuvier [Ursidae; Fel Selenarcti et Ursi], *Gardenia jasminoides* J. Ellis [Rubiaceae; Gardeniae Fructus], *Lonicera japonica* Thunb. [Caprifoliaceae; Lonicerae Japonicae Flos], *Bubalus bubalis* Linnaeus [Bovidae; Bubali Cornu], *Strobilanthes cusia* (Nees) Kuntze [Acanthaceae; Baphicacanthis cusiae rhizoma et radix], *Hyriopsis cumingii* (Lea) [Unionidae & Pteriidae; Margaritifera concha], *Sus scrofa domestica* Brisson. [Suidae; Suis Fellis Pulvis] | 20-40mL, Qd, ivgtt | Inadequate |

## 2 Extract and extraction process description of the TCMIs

| TCMIs | Extract and extraction process description |
| --- | --- |
| Reduning Injection | First, take 750g of Lonicerae Japonicae Flos, extract twice with 15 times and 10 times the amount of water respectively, each time heating at 90°C for 1 hour, and set aside the extract. Meanwhile, take 1250g of Artemisiae Annuae Herba, moisten with 4 times the amount of water, then perform steam distillation for 6 hours, and collect the volatile oil for later use. Combine the honeysuckle and sweet wormwood extracts, filter, and concentrate under reduced pressure to a relative density of 1.10 at 60°C. Then add ethanol to achieve an alcohol content of 75%, let stand for 24 hours, filter, and further concentrate the filtrate under reduced pressure to a relative density of 1.20 at 60°C. Adjust the pH to 2.0 using hydrochloric acid, then extract 10 times with an equal volume of ethyl acetate. Concentrate the extraction liquid under reduced pressure until no ethyl acetate odor remains, and vacuum dry to obtain dry honeysuckle and sweet wormwood extract powder. Next, take 600g of Gardeniae Fructus, crush into coarse powder, and reflux extract twice with 6 times the amount of 80% ethanol, each time for 1 hour. Combine the filtrates, recover the ethanol, and concentrate to a 1:1 ratio. Adjust the pH to 3.0 with hydrochloric acid, heat at 100°C for 1 hour, add solid gypsum equivalent to 1% of the crude drug weight, stir, refrigerate for 12 hours, and filter. Extract the filtrate 6 times with an equal volume of n-butanol, concentrate under reduced pressure until no n-butanol odor remains, and vacuum dry to obtain dry gardenia extract powder. In the preparation stage, take the above dry extract powders, add 1000 ml of water for injection, boil, add the dry extract powders and stir until uniform, boil for 4 minutes, cool to room temperature, refrigerate for 24 hours, and filter. Adjust the filtrate pH to 2 to 3, add 1% activated carbon, boil for 10 minutes, cool, refrigerate for 48 hours, filter, and add 0.5g of sodium bisulfite, stirring until uniform. Take 50 ml from the solution, heat to 30 to 40°C, add 2.0 to 8.0 ml of HS-15 and stir until uniform, add the volatile oil, combine the solutions and mix well. Process through ultrafiltration with molecular weight cut-offs of 30,000 and 10,000, and filter through a 0.22 μm microporous membrane to obtain the final product. |
| Xiyanping Injection | First, extract andrographolide from the andrographis herba as the raw material, mix and dissolve it with absolute ethanol in a certain proportion to form a clear solution. Then, under ice-water bath conditions maintaining a low temperature of 0 to 5°C, slowly add concentrated sulfuric acid dropwise with stirring to avoid local overheating and degradation. Subsequently, let the mixture stand at 10 to 25°C for 48 to 72 hours to complete the sulfonation reaction. After the reaction, add absolute ethanol to quench, maintaining the low temperature while slowly adding 20 to 40% sodium hydroxide solution dropwise to adjust the pH to 7.1 to 7.5 for neutralization. Next, adjust the ethanol content to 85 to 95%, refrigerate overnight at 0 to 5°C to precipitate by-products such as sodium sulfate, and filter to separate. Concentrate the filtrate under reduced pressure to recover ethanol until no alcohol odor remains, add water for injection to dissolve, refrigerate and filter to remove insoluble matter, then add 0.2 to 0.3% activated carbon, heat to boiling for 10 minutes for decolorization, and filter. Concentrate and dry to obtain the finished andrographolide sulfonate product. Subsequently, dissolve the sulfonate in water for injection, stir at 30 to 80°C until a clear solution is obtained. Add 0.1 to 0.2% activated carbon, stir for 10 minutes, filter for decolorization, repeat if necessary. Add water to the specified volume, adjust pH to 7.3 to 7.5 with 10% NaOH. Perform fine filtration, package, and sterilize at 105°C for 30 minutes to obtain the final injection product. |
| Yanhuning Injection | First, perform the esterification reaction: Mix andrographolide, succinic anhydride, pyridine, and anhydrous sodium sulfite in a molar ratio of 1:2 to 1:10. The amount of pyridine is 0.5 to 2 times the mass of andrographolide, and anhydrous sodium sulfite is 1% to 10% of the mass of andrographolide. Stir uniformly under a vacuum of 0.03 to 0.1 MPa, gradually heat to 50 to 100°C, and react for 1 to 2 hours. Dissolve the reaction product in 10 to 30 times its mass of hot water at 30 to 70°C, cool to -10 to 15°C for low-temperature crystallization, stir and let stand for 12 hours, then filter to obtain dehydrated andrographolide succinate hemiester. Next, perform the salt-forming reaction: First dissolve the hemiester in ≥95% ethanol, add a near-saturated potassium salt solution, heat to 70 to 100°C, pour into ≥95% ethanol, stir for 0.5 hours, let stand at room temperature for 3 to 4 hours, filter and dry to obtain potassium salt of dehydrated andrographolide succinate hemiester. Then dissolve the potassium salt in absolute ethanol, add a near-saturated sodium salt solution, heat to 70 to 100°C, pour into absolute ethanol, stir for 1 to 1.5 hours, let stand at room temperature for ≥12 hours, filter, wash the filter cake twice with absolute ethanol, and dry to obtain Yanhuning. Finally, prepare the injection: Dissolve sodium chloride in water to make a solution, add activated carbon, heat to boiling for 5 minutes, let stand and cool to 18 to 25°C, filter through a 0.45 μm microporous membrane and collect the filtrate. Add L-cysteine, citric acid, tartaric acid, and EDTA to the filtrate, dissolve, then adjust pH to 4 to 6 with 1 mol/L NaOH. Under nitrogen protection, add Yanhuning, stir to dissolve, add 0.02% activated carbon, heat to boiling for 2 minutes, coarsely filter while hot, then finely filter through a 0.22 μm microporous membrane, collect the filtrate under nitrogen, fill and seal vials, sterilize at 121°C for 8 minutes to obtain the finished Yanhuning Injection. |
| Tanreqing Injection | First, suspend bear bile powder in 8 to 12 times deionized water, add 8 to 12% solid sodium hydroxide, heat to boiling for saponification for 16 to 20 hours. Cool, adjust pH to 1.0 to 2.0 with dilute hydrochloric acid, filter to collect the precipitate, wash with water to neutral, dry at low temperature to obtain crude total ursodeoxycholic acid. Then reflux extract with activated carbon and ethyl acetate, filter, concentrate, cool for crystallization, vacuum dry to obtain purified total ursodeoxycholic acid. Next, add goat horn to 8 to 12 times 4 mol/L sulfuric acid, reflux heat for hydrolysis for about 16 hours, filter, wash the residue and combine the filtrates, adjust pH to 3.5 to 5.0 with lime milk, concentrate, decolorize with activated carbon, add ethanol for alcohol precipitation, refrigerate, ultrafilter, recover ethanol, concentrate and vacuum dry to obtain total amino acids. Then, crush scutellariae radix, decoct twice with 8 to 10 times water, combine the decoctions, adjust pH to 1.0 to 2.0 with hydrochloric acid, maintain temperature, let stand, and filter. Suspend the precipitate in water, adjust pH to 7.0 with sodium hydroxide, add an equal amount of ethanol to dissolve and filter, adjust pH to 1 to 3 with dilute hydrochloric acid, maintain temperature and let stand, coarsely filter, ultrafilter, wash with water, vacuum dry to obtain baicalin. Then, decoct Lonicerae Japonicae Flos twice with 10 to 15 times water, combine the filtrates and concentrate, adjust pH to 12 with lime milk, filter. Suspend the precipitate in ethanol, adjust pH to 3.0 to 4.0 with sulfuric acid, filter, adjust pH to 6.5 to 7.0 with sodium hydroxide, recover ethanol and concentrate, extract with n-butanol, recover solvent, dissolve in water, cool and let stand, coarsely filter, ultrafilter, concentrate and vacuum dry to obtain honeysuckle extract. Similarly, decoct forsythiae fructus twice with water, simultaneously collect volatile oil; combine decoctions and concentrate, add ethanol for alcohol precipitation, filter, recover ethanol, decolorize with activated carbon, extract with n-butanol, recover solvent, dissolve in water, cool and let stand, coarsely filter, ultrafilter, concentrate and vacuum dry to obtain forsythia extract. Finally, dissolve each of the above extracts separately in water for injection, adjust pH to 7.0 to 8.0, mix, add forsythia volatile oil and 10% propylene glycol, boil with activated carbon for 30 minutes, triple filter, add water for injection to make up to 1000 ml, readjust pH to 7.0 to 8.0, ultrafilter, filter through a 0.2 μm microporous membrane, fill, and sterilize at 121°C for 20 minutes to obtain the finished product. |
| Xuebijing Injection | First, process each herb separately: Take 100g of carthami flos, moisten with 30% ethanol for 6 hours, then percolate, collect the percolate and add 95% ethanol to achieve a concentration of 70%, refrigerate for 48 hours, filter, recover ethanol and concentrate to a thick paste, vacuum dry for later use. Take 100g of sliced paeoniae radix rubra, soak in 10 times purified water for 1 hour, decoct for 2 hours, combine the filtrates and concentrate, add egg white for precipitation, then add 95% ethanol to achieve 70% concentration, refrigerate for 24 hours, filter, recover ethanol, then extract four times with water-saturated n-butanol, combine the extracts, recover the solvent, dissolve the residue in water, refrigerate for 48 hours, filter, concentrate to a thick paste, and vacuum dry. Take 100g each of Chuanxiong rhizoma, salviae miltiorrhizae radix et rhizoma, and angelicae sinensis radix, similarly decoct twice with water, concentrate, add egg white for precipitation, add 95% ethanol to 70% concentration, refrigerate and filter, recover ethanol, add activated carbon for decolorization, then extract with water-saturated n-butanol, and treat the residue similarly to red peony. Subsequently, dissolve each of the above semi-finished products in water separately, filter, combine, adjust pH to 6.5 to 7.0, add 95% ethanol to 80% concentration, refrigerate for 24 hours, filter, and recover ethanol. Add 4.5% injection-grade glucose dissolved in water for injection, add water to make up to 1000ml, adjust pH, add activated carbon, boil for 15 minutes, cool, purge with nitrogen, and filter to clarity. Quantitatively fill into ampoules, purge with nitrogen and seal, sterilize with flowing steam at 100°C for 30 minutes. |
| Shuanghuanglian Injection | Decoct scutellariae radix twice with water, each time for 1 hour, filter, combine the filtrates, adjust pH to 1.0 to 2.0 with 2mol/L hydrochloric acid solution, maintain at 80°C for 30 minutes, let stand for 12 hours, filter. Add 8 times the amount of water to the precipitate, stir, adjust pH to 7.0 with 10% sodium hydroxide solution, add an equal amount of ethanol, stir to dissolve the precipitate, filter. Adjust the filtrate pH to 2.0 with 2mol/L hydrochloric acid solution, maintain at 60°C for 30 minutes, let stand for 12 hours, filter. Wash the precipitate with ethanol to pH 4.0, add 10 times the amount of water, stir, adjust pH to 7.0 with 10% sodium hydroxide solution. Add 5g of activated carbon per 1000ml of solution, stir thoroughly, maintain at 50°C for 30 minutes, add an equal amount of ethanol, stir uniformly, filter. Adjust the filtrate pH to 2.0 with 2mol/L hydrochloric acid solution, maintain at 60°C for 30 minutes, let stand for 12 hours, filter. Wash the precipitate with a small amount of ethanol, dry below 60°C, and set aside. Lonicerae Japonicae Flos and Forsythiae fructus separately in warm water for 30 minutes, then decoct twice, each time for 1 hour, filter, combine the filtrates, concentrate to a relative density of 1.20 to 1.25, cool to 40°C, slowly add ethanol to achieve an alcohol content of 75%, stir thoroughly, let stand for more than 12 hours, collect the supernatant, recover ethanol until no alcohol odor remains, add 4 times the amount of water, let stand for more than 12 hours, collect the supernatant, concentrate to a relative density of 1.10 to 1.15, cool to 40°C, add ethanol to achieve an alcohol content of 85%, let stand for more than 12 hours, collect the supernatant, recover ethanol until no alcohol odor remains, and set aside. Take the baical skullcap root extract, add an appropriate amount of water, heat, adjust pH to 7.0 with 10% sodium hydroxide solution to dissolve, add the above honeysuckle and forsythia extracts, add water to 1000ml, add 5g of activated carbon, adjust pH to 7.0, heat to boiling and maintain gentle boiling for 15 minutes, cool, filter, add water for injection to 1000ml, sterilize, refrigerate, filter, concentrate, freeze-dry to make powder, and package. Alternatively, take the baical skullcap root extract, add an appropriate amount of water, heat, adjust pH to 7.0 with 10% sodium hydroxide solution to dissolve, add the above honeysuckle and forsythia extracts and an appropriate amount of water for injection, add 5g of activated carbon per 1000ml of solution, adjust pH to 7.0, heat to boiling and maintain gentle boiling for 15 minutes, cool, filter, sterilize, filter, fill, freeze-dry, and cap to obtain the product. |
| Qingkailing Injection | Decoct Isatidis Radix twice with water, each time for 1 hour, combine the decoctions, filter, concentrate the filtrate to 200ml, add ethanol to achieve an alcohol content of 60%, refrigerate, filter, recover ethanol from the filtrate, add water, refrigerate for later use. Decoct Gardeniae Fructus twice with water, the first time for 1 hour, the second time for 0.5 hours, combine the decoctions, filter, concentrate the filtrate to 25ml, add ethanol to achieve an alcohol content of 60%, refrigerate, filter, recover ethanol from the filtrate, add water, refrigerate for later use. Decoct Lonicerae Japonicae Flos twice with water, each time for 0.5 hours, combine the decoctions, filter, concentrate the filtrate to 60ml, add ethanol to achieve an alcohol content of 75%, filter, adjust the filtrate pH to 8.0, refrigerate, recover ethanol, then add ethanol again to achieve an alcohol content of 85%, refrigerate, filter, recover ethanol from the filtrate, add water, refrigerate for later use. Hydrolyze buffalo horn powder with barium hydroxide solution and pearl mother-of-pearl powder with sulfuric acid respectively for 7 to 9 hours, filter, combine the filtrates, adjust pH to 3.5 to 4.0, filter, add ethanol to the filtrate to achieve an alcohol content of 60%, refrigerate, filter, recover ethanol from the filtrate, add water, refrigerate for later use. Combine the gardenia liquid, indigowoad root liquid, and the mixed hydrolysate of buffalo horn and pearl mother-of-pearl, then add to a 75% ethanol solution containing cholic acid and hyodeoxycholic acid, mix well, add ethanol to achieve an alcohol content of 75%, adjust pH to 7.0, refrigerate, filter, recover ethanol from the filtrate, add water, refrigerate for later use. Dissolve baicalin in water for injection, adjust pH to 7.5, add the honeysuckle extract liquid, mix well, combine with the above various reserve liquids, mix well, add water for injection to 1000ml, then treat with activated carbon, refrigerate, fill and seal, sterilize to obtain the product. |

**3 The safety characteristics of the treatment groups**

| Treatment group | Adverse reaction | Drug interactions and incompatibility contraindications |
| --- | --- | --- |
| Reduning Injection | Nausea and vomiting, abdominal pain and diarrhea, rash, leukopenia, liver function damage, etc. | Mixing with penicillins, aminoglycosides, macrolides, etc., may produce precipitation or turbidity. |
| Xiyanping Injection | Palpitations, digestive tract reactions, allergic reactions, dizziness and headache, irritability, hemolytic anemia, decreased skin oxygenation, drug-induced fever, etc. | It is strictly prohibited to use this drug together with other medications in the same container. |
| Yanhuning Injection | Allergic reactions, pyrogen-like reactions, gastrointestinal reactions, dizziness and headache, liver function damage, etc. | Avoid combining with acidic or alkaline drugs or those containing sodium bisulfite or sodium metabisulfite as antioxidants. It is not suitable for combination with aminoglycoside or quinolone drugs. |
| Tanreqing Injection | Digestive tract reactions, dizziness and headache, rash, leukopenia, allergic reactions, etc. | Must not be mixed with other drugs for intravenous infusion |
| Xuebijing Injection | Nausea and vomiting, abdominal pain and diarrhea, rash, rizziness and headache, dry cough, bleeding, etc. | During the intravenous infusion process, it is prohibited to be mixed with other injectables. |
| Shuanghuanglian Injection | Chills, fever, rapid breathing, chest tightness, dizziness, rash, etc. | It is prohibited to use in combination with aminoglycosides (such as amikacin, gentamicin, kanamycin, streptomycin, etc.), macrolides (such as erythromycin, clarithromycin, etc.), quinolones (such as ciprofloxacin, etc.), penicillins, etc. |
| Qingkailing Injection | Rash, shortness of breath, dizziness, nausea, diarrhea, drug fever, etc. | It can not be used in combination with gentamicin sulfate, penicillin G potassium, penicillin G, epinephrine, alamin, dopamine lactamate, magnesium sulfate injection, hamidonine, and mefenbutamine sulfate. |
