# Supplementary material for: Therapeutic effects of traditional Chinese medicine injections with heat-clearing and detoxifying properties on viral pneumonia: a systematic review and network meta-analysis
Source: Front Pharmacol. 2026 May 14;17:1771777. doi: 10.3389/fphar.2026.1771777 (PMC13216718; doi:10.3389/fphar.2026.1771777)
Supplement: Supplementary file 4 [file Supplementaryfile3.docx]

**Supplementary Material 3:** Basic characteristics of included literature.

| Study | Sample size | | Sex | | Age (years) | Treatment | | Course (days) | Clinical outcomes | Main content |
| --- | --- | --- | --- | --- | --- | --- | --- | --- | --- | --- |
|  | T | C | M | F |  | T | C |  |  |  |
| Zou and Wang (2000) | 40 | 40 | 48 | 32 | 0.10 ~ 1.5 | SHL 10 ~ 15 mL·kg^-1^·d^-1^ Qd + CM | CM | NA | b, c, g, i | SHL + CM was significantly superior to CM in terms of antipyretic time, cough disappearance time, disappearance time of lung rales, and average hospital stay. |
| Qin and Wang (2003) | 30 | 30 | 34 | 26 | 0.5 ~ 4 | SHL 0.6 mg Bid + CM | CM | 5 ~ 7 | a, b, c, g, h | SHL + CM had significantly shorter antipyretic time, cough cessation time, asthma relief time, and disappearance time of lung rales compared to CM, but the difference in total effective rate was not statistically significant. |
| Hu and Zhu (2006) | 38 | 34 | 46 | 26 | T: 20 ± 10  C: 24 ± 10 | TRQ 20 mL Qd + CM | CM (antiviral drugs and so on) | 10 | a | TRQ + CM had a higher total effective rate than CM. |
| Yang (2007) | 60 | 56 | 62 | 54 | T: 0.5 ~ 8  C: 0.67 ~ 7 | YHN 10 mg·kg^-1^·d^-1^ Qd + CM | CM (antiviral drugs and so on) | 7 ~ 14 | a, b, c, g | YHN + CM significantly shortened antipyretic time, cough cessation time, and disappearance time of lung rales, and improved clinical efficacy. |
| Huang (2008) | 30 | 30 | 31 | 29 | < 18 | YHN 5 ~ 10 mg·kg^-1^·d^-1^ + CM | CM (antiviral drugs and so on) | NA | b, c, g, h, i | YHN + CM was superior to CM in terms of antipyretic, cough cessation, asthma relief, disappearance of lung rales, and hospital stay. |
| Wang et al. (2008) | 60 | 57 | 55 | 62 | T: ≤ 6  C: ≤ 11 | YHN 5 ~ 15 mg·kg^-1^·d^-1^ Qd + CM | CM (antiviral drugs and so on) | 7 | a, b, c, g, i | YHN + CM shortened antipyretic time, cough disappearance time, disappearance time of lung rales, and average hospital stay. |
| Guo (2009) | 40 | 40 | 37 | 43 | T: 0.17 ~ 11  C: 0.25 ~ 12 | XYP 5 ~ 10 mg Qd + CM | CM (antiviral drugs and so on) | 5 ~ 7 | b, g, h, i | XYP + CM was significantly superior to CM in terms of fever resolution, asthma, disappearance of lung rales, and days to recovery. |
| Li (2010) | 100 | 100 | 114 | 86 | T: 1.125  C: 1.175 | YHN 10 mg·kg^-1^·d^-1^ Qd + CM | CM (glucocorticoids and so on) | 7 | a | YHN + CM had a significantly higher total effective rate than CM. |
| Liu et al. (2010a) | 72 | 58 | 61 | 69 | T: 0.67 ~ 7  C: 0.75 ~ 7 | QKL 10 ~ 20 mL Qd + CM | CM (antiviral drugs and so on) | NA | a | QKL + CM had a significantly higher total effective rate than CM. |
| Liu et al. (2010b) | 50 | 50 | 53 | 47 | T: 0.75 ~ 6  C: 0.83 ~ 6 | YHN 80 ~ 160 mg Qd + CM | CM (antiviral drugs and so on) | NA | a, b, c, g | YHN + CM significantly shortened antipyretic time, cough disappearance time, and disappearance time of lung rales, and increased the total effective rate. |
| Wang (2010) | 85 | 85 | 103 | 67 | T: 41.3  C: 42 | SHL 2.4 g·d^-1^ + CM | CM (antiviral drugs and so on) | NA | a | SHL + CM had a significantly higher total effective rate than CM. |
| Li (2011) | 50 | 50 | 55 | 45 | T: 0.75 ~ 5.5  C: 0.67 ~ 5 | YHN 80 ~ 160 mg Qd + CM | CM (antiviral drugs and so on) | NA | a | YHN + CM had a significantly higher total effective rate than CM. |
| Xiang et al. (2011) | 46 | 40 | 41 | 45 | T: 4.03 ± 1.17  C: 4.07 ± 1.12 | RDN 0.6 mL·kg^-1^·d^-1^ + CM | CM | 5 ~ 7 | a, b, g | RDN + CM shortened antipyretic time and increased the total effective rate. |
| Ye and Su (2011) | 30 | 30 | NA | NA | 0.83 ~ 7 | TRQ 0.3 ~ 0.5 mL·kg^-1^·d^-1^ Qd + CM | CM (antiviral drugs and so on) | 7 | b, c, g, i | TRQ + CM was significantly superior to CM in terms of antipyretic time, cough disappearance time, disappearance time of lung rales, and hospital stay. |
| Cao and Xue (2012) | 34 | 34 | 48 | 20 | T: 48.3 ± 8.7  C: 47.2 ± 7.9 | TRQ 20 mL Qd + CM | CM (antiviral drugs and so on) | 14 | a, b | TRQ + CM significantly increased the total effective rate and shortened the time for body temperature to return to normal. |
| Cheng (2012) | 120 | 120 | 104 | 136 | 4 ~ 8 | SHL 30 mg·kg^-1^·d^-1^ Qd + CM | CM (antiviral drugs and so on) | NA | a, b, c, g | SHL + CM had significantly shorter antipyretic time, cough disappearance time, and disappearance time of lung rales compared to CM. |
| Gao (2012) | 78 | 78 | 96 | 60 | 0.33 ~ 3 | XYP 0.4 mL·kg^-1^·d^-1^ + CM | CM (antiviral drugs and so on) | 5 ~ 7 | a | XYP + CM had a significantly higher total effective rate than CM. |
| Liu et al. (2012) | 67 | 53 | 58 | 62 | T: 0.75 ~ 7  C: 0.67 ~ 7 | YHN 5 ~ 10 mg·kg^-1^·d^-1^ Qd + CM | CM (antiviral drugs and so on) | 5 ~ 7 | a, b, c, g, f | YHN + CM significantly shortened antipyretic time, cough disappearance time, and disappearance time of lung rales, and increased the total effective rate. |
| Wang and Jin (2012) | 61 | 61 | 63 | 59 | T: 2.98 ± 0.61  C: 2.52 ± 1.82 | XYP 5 ~ 10 mg·kg^-1^·d^-1^ + CM | CM (antiviral drugs and so on) | 5 ~ 7 | a, b, g, h | XYP + CM had a significantly higher total effective rate than CM; XYP + CM showed faster onset of action in improving symptoms such as asthma and lung rales. |
| Yang (2012) | 43 | 43 | 45 | 41 | T: 41.0 ± 2.2  C: 39.0 ± 3.6 | SHL 10 mL·kg^-1^ Qd + CM | CM (antiviral drugs and so on) | 5 | a | SHL + CM had a higher total effective rate than CM. |
| Jiang (2013) | 50 | 50 | 53 | 47 | T:0.75 ~ 6  C: 0.83 ~ 6 | YHN 80 ~ 160 mg Qd + CM | CM (antiviral drugs and so on) | NA | a, b, c, g | YHN + CM had significantly shorter antipyretic time, cough disappearance time, and disappearance time of lung rales compared to CM. |
| Li and Wu (2013) | 32 | 32 | 38 | 26 | 57.52 ± 12.90 | XYP 30 mL Qd + CM | CM | 7 | a, b | XYP + CM significantly improved clinical efficacy and shortened the time for body temperature to return to normal. |
| Li (2013) | 63 | 35 | 59 | 39 | 3.1 ± 0.4 | YHN 20 ~ 160 mg Qd + CM | CM (antiviral drugs and so on) | 7 ~ 10 | a, b, c, g | YHN + CM significantly shortened the disappearance time of main clinical symptoms such as fever, cough, and lung rales, and improved clinical efficacy. |
| Qing (2013) | 45 | 45 | 57 | 33 | T: 5.1 ± 1.2  C: 4.8 ± 1.1 | SHL 30 mg·kg^-1^·d^-1^ Qd + CM | CM (antiviral drugs and so on) | 7 | a, b, c, g, j, k | SHL + CM significantly increased the total clinical effective rate and shortened the disappearance time of cough, lung rales, fever, etc. |
| Shi and Yan (2013) | 25 | 25 | 23 | 27 | T: 0.75 ~ 8  C: 0.67 ~ 10 | YHN 10 mg·kg^-1^·d^-1^ Qd + CM | CM (antiviral drugs and so on) | 7 | a, b, c, g | YHN + CM had shorter antipyretic time, cough disappearance time, and disappearance time of lung rales compared to CM. |
| Zhao (2013) | 40 | 40 | 51 | 29 | T: 4.26 ± 1.37  C: 4.13 ± 1.28 | TRQ 0.3 ~ 0.5 mL·kg^-1^ Qd + CM | CM (antiviral drugs and so on) | 7 | a, b, c, g | TRQ + CM had a higher total effective rate and significantly shortened the disappearance time of symptoms such as fever, cough, and rales. |
| Kang (2015) | 126 | 108 | NA | NA | T: 18 ~ 45  C: 20 ~ 50 | XYP 3 mL Qd + CM | CM (antiviral drugs and so on) | 7 | a | XYP + CM had a higher total effective rate than CM. |
| Lin (2015) | 36 | 30 | 44 | 22 | 36.6 ± 5.4 | RDN 0.6 mL·kg^-1^·d^-1^ Qd + CM | CM (immunoglobulin and so on) | 5 | b, c, g, h, j, k, f | RDN + CM significantly shortened patients' antipyretic time, cough disappearance time, and rales resolution time, improved T lymphocyte subset ratios, and enhanced immune function. |
| Xue (2015) | 38 | 38 | 42 | 34 | T: 0.17 ~ 2.33  C: 0.25 ~ 2.17 | TRQ 0.3 mL·kg^-1^ Qd + CM | CM (antiviral drugs and so on) | NA | b, c, g, h, f | TRQ + CM significantly shortened the disappearance time of symptoms such as fever, wheezing, cough, and lung rales, with superior efficacy compared to CM. |
| Zhang and Zhou (2015) | 153 | 152 | 161 | 144 | T: 2.3 ± 0.8  C: 2.4 ± 0.9 | XYP 10 mg·kg^-1^·d^-1^ Qd + CM | CM (antiviral drugs and so on) | 7 | a | XYP + CM had a higher total effective rate than CM. |
| Luo (2016) | 45 | 45 | 53 | 37 | T: 0.42 ~ 1.08  C: 0.5 ~ 1 | TRQ 0.3 ~ 0.5 mL·kg^-1^ Qd + CM | CM (antiviral drugs and so on) | NA | a, c, g, h, i, f | TRQ + CM had significantly shorter disappearance times for lung rales, cough, wheezing, and hospital stay compared to CM. |
| Wang et al. (2016) | 60 | 60 | NA | NA | < 18 | XYP 5 mg·kg^-1^·d^-1^ + CM | CM (antiviral drugs and so on) | 7 | a, b, c, g, d, e, n, f | XYP + CM significantly shortened the disappearance time of cough, fever, and lung rales, increased the total effective rate, and more effectively reduced inflammatory factor levels. |
| Wu et al. (2016) | 46 | 46 | 53 | 40 | 54.24 ± 10.22 | XBJ 50 mL Bid + CM | CM (immunoglobulin and so on) | 7 | a, b, c, h, d, e, o | XBJ + CM had significantly shorter antipyretic time, cough resolution time, and wheezing resolution time compared to CM; after treatment, hs-CRP, IL-6, and TNF-伪 levels decreased significantly in both groups, but the decrease was more significant in the XBJ + CM group. |
| Xiao (2016) | 50 | 50 | 53 | 47 | T: 0.5 ~ 3.17  C: 0.58 ~ 3.33 | XYP 5 mg·kg^-1^·d^-1^ + CM | CM (immunoglobulin and so on) | 3 ~ 5 | a | XYP + CM had a significantly higher total effective rate than CM. |
| Yin et al. (2016) | 34 | 33 | NA | NA | T: 57.9 ± 4.3  C: 59.2 ± 4.6 | TRQ 20 mL Bid + CM | CM (antiviral drugs and so on) | 14 | a, b, c, h | TRQ + CM was superior to CM in shortening the improvement time of clinical symptoms (cough, wheezing, fever, etc.). |
| Zhao et al. (2016) | 50 | 50 | 62 | 38 | T: 3.3 ± 1.1  C: 3.5 ± 1.2 | RDN 0.6 mL·kg^-1^·d^-1^ Qd + CM | CM (immunoglobulin and so on) | 5 | a, b, c, g, j, o, f | RDN + CM significantly increased the total effective rate and shortened the time for fever resolution, cough disappearance, dyspnea disappearance, and lung rales disappearance, and enhanced immune function. |
| Chen (2017) | 53 | 53 | 55 | 51 | T: 2.9 ± 1.3  C: 3.0 ± 1.2 | YHN 10 mg Qd + CM | CM (antiviral drugs and so on) | 7 | a, b, c, g | YHN + CM significantly shortened cough disappearance time, disappearance time of lung rales, and antipyretic time. |
| Guo (2017) | 32 | 32 | 35 | 29 | T: 3.4 ± 2.2  C: 3.8 ± 2.1 | XYP 0.3 mg·kg^-1^·d^-1^ + CM | CM (antiviral drugs and so on) | 10 | a | XYP + CM had a significantly higher total effective rate than CM. |
| Li et al. (2017) | 53 | 52 | 62 | 43 | T: 3.05 ± 0.83  C: 3.14 ± 0.79 | QKL 6 ~ 10 mL Qd + CM | CM (antiviral drugs and so on) | 7 | a, b, c, g, d, e, n | QKL + CM significantly increased the total clinical effective rate, more effectively reduced inflammatory factor levels, and shortened the disappearance time of fever, cough, and lung rales. |
| Li (2017) | 35 | 35 | NA | NA | T: 6.88 ± 1.46  C: 7.12 ± 1.34 | XYP 10 mg·kg^-1^·d^-1^ Qd + CM | CM (antiviral drugs and so on) | 7 | a | XYP + CM had a significantly higher total effective rate than CM. |
| Liu et al. (2017) | 60 | 68 | 75 | 53 | T: 26.2 ± 8.7  C: 25.8 ± 9.1 | XYP 5 ~ 10 mg·kg^-1^·d^-1^ Qd + CM | CM (antiviral drugs and so on) | 7 | a, f | XYP + CM showed significantly superior efficacy compared to CM, with a lower incidence of adverse reactions. |
| Luo (2017) | 47 | 45 | 43 | 49 | T: 7.51 ± 3.52  C: 7.42 ± 3.56 | RDN 20 mL·d^-1^ + CM | CM (antiviral drugs and so on) | 5 | b, i, f | RDN + CM improved fever resolution more quickly without increasing the incidence of adverse reactions. |
| Zeng (2018) | 31 | 31 | 43 | 19 | T: 3.4 ± 0.3  C: 3.5 ± 0.9 | RDN 0.6 mL·kg^-1^ Qd + CM | CM (antiviral drugs and so on) | 5 | a, b, c, g, h, f | RDN + CM significantly increased the total clinical effective rate, shortened the resolution time of symptoms and signs such as fever, cough, wheezing, and lung rales, without increasing the incidence of adverse reactions. |
| Zhai (2018) | 44 | 44 | 54 | 34 | T: 4.5 ± 2.5  C: 4.2 ± 2.6 | RDN 0.6 mL·kg^-1^·d^-1^ Qd + CM | CM (antiviral drugs and so on) | 5 | j, k | RDN + CM showed more significant improvement in immune function. |
| Gao et al. (2018) | 61 | 61 | 66 | 56 | T: 5.5 ± 1.7  C: 5.7 ± 1.6 | XYP 5 ~ 10 mg·kg^-1^ Qd + CM | CM (antiviral drugs and so on) | 21 | a, d, e, f | XYP + CM more effectively reduced serum inflammatory factor levels. |
| Jing (2018) | 150 | 150 | 152 | 148 | T: 47.5 ± 1.4  C: 47.2 ± 1.6 | XBJ 50 mL Tid + CM | CM (immunoglobulin and so on) | 30 | d, e, o | XBJ + CM more effectively reduced inflammatory factor levels. |
| Liang (2018) | 39 | 39 | 41 | 37 | T: 0.17 ~ 1.92  C: 0.08 ~ 1.83 | TRQ 0.4 mL·kg^-1^ Qd + CM | CM (antiviral drugs and so on) | 7 | a, b, c, g, h, f | TRQ + CM significantly improved clinical efficacy, accelerated the normalization time of symptoms such as fever, cough, and wheezing, without increasing the incidence of adverse reactions. |
| Liu (2018) | 98 | 98 | 101 | 95 | T: 2.3 ± 1.5  C: 2.4 ± 1.5 | TRQ 0.3 ~ 0.5 mg·kg^-1^ Qd + CM | CM (antiviral drugs and so on) | 7 | a, b, c, g, h, i, f | TRQ + CM significantly increased the total effective rate and shortened the disappearance time of symptoms such as cough, fever, wheezing, and hospital stay. |
| Song and Yang (2018) | 60 | 60 | 62 | 58 | T: 1.62 ± 0.46  C: 1.54 ± 0.41 | XYP 0.2 ~ 0.4 mL·kg^-1^·d^-1^ + CM | CM | NA | d, e, n | XYP + CM significantly reduced serum inflammatory factor levels. |
| Yu (2018) | 31 | 31 | 33 | 29 | T: 5.4 ± 1.7  C: 5.5 ± 1.3 | RDN 20 mL·d^-1^ + CM | CM (antiviral drugs and so on) | 5 | a | RDN + CM had a significantly higher total effective rate than CM. |
| Zhang (2018) | 50 | 50 | 55 | 45 | T: 50 ~ 69  C: 49 ~ 68 | TRQ 20 mL Bid + CM | CM (antiviral drugs and so on) | 7 | a, b, c, g, h | TRQ + CM more rapidly improved clinical symptoms such as fever, cough, wheezing, and lung rales, with a higher total effective rate. |
| Zhang et al. (2018) | 50 | 52 | 47 | 55 | < 18 | XYP 2.5 mg·kg^-1^ Tid + CM | CM (glucocorticoids and so on) | 7 | a, c, h, f | XYP + CM had significantly shorter cough disappearance time and wheezing relief time compared to CM. |
| Zhao et al. (2018) | 70 | 70 | 69 | 71 | T: 5.78 ± 1.67  C: 5.05 ± 1.34 | RDN ≤ 10 mL·d^-1^ Qd + CM | CM (antiviral drugs and so on) | 5 ~ 7 | a, d, n | RDN + CM increased the total effective rate and reduced inflammatory factor levels. |
| Wang and Liu (2019) | 44 | 44 | 47 | 41 | T: 0.5 ~ 1  C: 0.5 ~ 1.08 | TRQ 0.3 ~ 0.5 mL·kg^-1^ Qd + CM | CM (antiviral drugs and so on) | 7 | a | TRQ + CM had a significantly higher total effective rate than CM. |
| Zhang (2019) | 120 | 120 | 131 | 109 | T: 57.51 ± 4.13  C: 58.04 ± 4.89 | TRQ 20 mL Bid + CM | CM (antiviral drugs and so on) | NA | a, b, c, g, h | TRQ + CM significantly shortened the resolution time of symptoms such as fever, cough, wheezing, and rales, and increased the treatment effective rate. |
| Fan et al. (2020) | 80 | 80 | 78 | 82 | T: 4.79 ± 0.53  C: 4.81 ± 0.46 | YHN 0.4 g·d^-1^ + CM | CM (antiviral drugs and so on) | 5 | a, k, f | YHN + CM demonstrated significant clinical efficacy and improved immune function. |
| Jiang and Zuo (2020) | 30 | 30 | 33 | 27 | T: 5.32 ± 0.17  C: 4.58 ± 0.29 | RDN 0.6 mL·kg^-1^·d^-1^ Qd + CM | CM (immunoglobulin and so on) | NA | b, c, g | RDN + CM more effectively shortened the resolution time of clinical symptoms. |
| Lan and Yan (2020) | 67 | 67 | 65 | 59 | T: 1.35 ± 0.65  C: 1.42 ± 0.37 | RDN 0.6 mL·kg^-1^ + CM | CM (antiviral drugs and so on) | 7 | a, b, c, g, h | RDN + CM significantly increased the total clinical effective rate and shortened the improvement time for clinical symptoms such as fever, cough, wheezing, and lung rales. |
| Li and Pan (2020) | 51 | 51 | 57 | 45 | 4.68 ± 2.51 | YHN 160 ~ 400 mg Qd + CM | CM (antiviral drugs and so on) | 6 | b, c, g | YHN + CM significantly shortened the resolution time of clinical symptoms such as cough and improved treatment outcomes. |
| Li and Zhang (2020) | 60 | 60 | 69 | 51 | T: 50.00 ± 3.50  C: 49.50 ± 3.00 | XBJ 50 mL Bid + CM | CM (immunoglobulin and so on) | 7 | a, b, c, g, d, l, m, f | XBJ + CM showed significant efficacy, effectively improving immune function, reducing inflammatory response, accelerating symptom relief, and decreasing the incidence of adverse reactions. |
| Lin et al. (2020) | 34 | 34 | 32 | 36 | T: 3.43 ± 1.87  C: 3.24 ± 1.80 | YHN 0.16 ~ 0.4 g·d^-1^ Qd + CM | CM (antiviral drugs and so on) | 7 | a, b, c, g, d, e, f | YHM + CM more effectively relieved symptoms, reduced inflammatory response, and improved efficacy. |
| Qiao and He (2020) | 55 | 55 | 63 | 47 | T: 4.69 ± 0.28  C: 4.71 ± 0.35 | TRQ 0.5 mL·kg^-1^ Qd + CM | CM (antiviral drugs and so on) | 14 | a, b, c, g, i, d, e, n, o | TRQ + CM significantly increased the total effective rate, shortened antipyretic time, cough disappearance time, lung rales disappearance time, and hospital stay, and more effectively reduced serum inflammatory factor levels. |
| Qin et al. (2020) | 21 | 26 | 23 | 24 | T: 58.0 ± 2.9  C: 58.3 ± 2.9 | RDN 20 mL Qd + CM | CM (glucocorticoids and so on) | 5 ~ 7 | i, k, e | RDN + CM significantly reduced inflammatory factor levels and shortened hospital stay. |
| Zhang et al. (2020) | 22 | 22 | 22 | 22 | T: 49.05 ± 14.19  C: 45.95 ± 14.68 | XBJ 100 mL Bid + CM | CM (antiviral drugs and so on) | 7 | a, f | XBJ + CM had a significantly higher total effective rate than CM. |
| Hu et al. (2021) | 49 | 48 | 52 | 45 | T: 4.52 ± 1.23  C: 4.13 ± 1.58 | YHN 10 mg·kg^-1^ Qd + CM | CM (antiviral drugs and so on) | 5 | k, o, f | YHN + CM significantly reduced serum inflammatory factor levels, alleviated inflammatory response, and improved immune function. |
| Li and Xiong (2021) | 27 | 27 | 30 | 24 | T: 3.39 ± 1.94  C: 3.24 ± 1.80 | RDN 0.5 ~ 0.6 mL·kg^-1^·d^-1^ (≤ 10 mL) Qd + CM | CM (antiviral drugs and so on) | 7 | a, b, c, g | RDN + CM significantly shortened fever resolution time, cough disappearance time, and rales disappearance time, and increased the clinical treatment effective rate. |
| Su (2021) | 57 | 56 | 60 | 53 | T: 53.1 ± 6.9  C: 53.7 ± 6.5 | XBJ 50 mL Bid + CM | CM (immunoglobulin and so on) | 7 | b, c, h, d, l, m, e, o | XBJ + CM effectively regulated immune function and accelerated symptom relief. |
| Xu (2021) | 56 | 56 | 69 | 43 | T: 6.21 ± 0.22  C: 6.13 ± 0.40 | TRQ 0.5 mL·kg^-1^ Qd + CM | CM (antiviral drugs and so on) | 14 | a, b, c, g | TRQ + CM significantly increased the total effective rate, shortened antipyretic time, cough disappearance time, and lung rales disappearance time. |
| Zuo (2021) | 50 | 50 | 50 | 50 | 4.43 ± 1.28 | RDN 0.2 ~ 0.4 mL·kg^-1^·d^-1^ + CM | CM (antiviral drugs and so on) | 5 | a, f | RDN + CM had a significantly higher total clinical effective rate than CM. |
| Zhang et al. (2021) | 65 | 65 | 32 | 28 | T: 44.31 ± 13.45  C: 48.25 ± 14.22 | XYP 10 mg·kg^-1^ Qd + CM | CM (antiviral drugs and so on) | 7 ~ 14 | b | XYP + CM significantly shortened patients' antipyretic time. |
| Gao et al. (2022) | 43 | 43 | 48 | 38 | T: 51.51 ± 5.73  C: 52.52 ± 6.08 | XBJ 50 mL Bid + CM | CM (antiviral drugs and so on) | 7 | b, c, g, h, d, j, k, f | XBJ + CM significantly shortened the disappearance time of symptoms and signs such as fever, cough, and wheezing, improved patients' immune function, and reduced serum inflammatory factors. |
| Luo et al. (2022) | 45 | 45 | 51 | 39 | T: 5.80 ± 1.46  C: 5.49 ± 1.22 | RDN 0.5 mL·kg^-1^ Qd + CM | CM (antiviral drugs and so on) | 7 | a, b, c, g, i, d, f | RDN + CM accelerated symptom relief, enhanced immunity, reduced inflammation, and had a good safety profile. |
| Mo et al. (2022) | 41 | 41 | 53 | 39 | T: 42.56 ± 7.83  C: 44.61 ± 5.96 | XYP 0.5 mL·kg^-1^ Qd + CM | CM (antiviral drugs and so on) | 7 | a, b, g, h, d, j, k, e, n, f | XYP + CM treatment was safe and effective, with a mechanism possibly related to reducing inflammatory response and enhancing immunity. |
| Chang et al. (2023) | 41 | 41 | 41 | 41 | T: 3.21 ± 0.40  C: 3.02 ± 0.45 | YHN 5 ~ 10 mg·kg^-1^·d^-1^ Qd + CM | CM (antiviral drugs and so on) | 7 | a, b, c, g, i, l, m, o, f | YHN + CM had higher efficacy than CM; during the same period, YHN + CM resulted in shorter disappearance times for clinical fever, cough, dyspnea, and lung rales compared to CM, accelerating recovery, and hospital stay was shorter than CM; inflammatory factor levels in the YHN + CM group were lower than in the CM group. |
| Gao and Du (2023) | 49 | 49 | 51 | 47 | T: 45.65 ± 5.56  C: 45.65 ± 5.56 | RDN 20 mL Qd + CM | CM (antiviral drugs and so on) | 5 | b, c, h, e, o, f | RDN + CM promoted symptom recovery, reduced inflammatory response, and had an ideal safety profile. |
| Guo and Huang (2023) | 20 | 20 | 27 | 13 | T: 65  C: 63 | TRQ 30 mL Qd + CM | CM | 7 | k | TRQ + CM showed significant relief of inflammation. |
| Zhang et al. (2023) | 36 | 41 | 46 | 31 | T: 83.25 ± 12.22  C: 79.43 ± 13.12 | RDN 20 mL Qd + CM | CM (antiviral drugs, glucocorticoids, and so on) | 7 | i | RDN + CM effectively shortened hospital stay. |
| Li (2024) | 51 | 51 | 53 | 29 | T: 45.08 ± 5.46  C: 45.62 ± 6.06 | RDN 20 mL Qd + CM | CM (antiviral drugs and so on) | 5 | a, b, c, g, h, f | RDN + CM had a higher total effective rate and shorter clinical symptom disappearance time. |
| Liu (2024) | 30 | 30 | 33 | 27 | T: 34.53 ± 10.21  C: 33.59 ± 9.97 | XYP 500 mg Qd + CM | CM (antiviral drugs and so on) | 5 | b, c, f | XYP + CM shortened the relief time of clinical symptoms. |
| Wang et al. (2024) | 38 | 36 | 42 | 32 | T: 65.53 ± 8.04  C: 64.21 ± 8.02 | XBJ 50 mL Bid + CM | CM (antiviral drugs and so on) | 14 | a, b, c, i, e, f | XBJ + CM effectively improved pulmonary inflammatory response and accelerated recovery. |
| Mao et al. (2024) | 40 | 40 | 44 | 36 | T: 42.50 ± 3.32  C: 42.28 ± 3.62 | YHN 400 mg Qd + CM | CM (antiviral drugs and so on) | 5 | a, b, c, d, f | YHN + CM more effectively relieved symptoms with a good safety profile. |
| Cai (2025) | 39 | 39 | 44 | 34 | T: 3.19 ± 0.10  C:3.22 ± 0.14 | XYP 5 ~ 10 mg·kg^-1^ Qd + CM | CM (antiviral drugs and so on) | 5 | a, b, c, g, i, d, e, o, f | XYP + CM increased the total effective rate, shortened clinical symptom disappearance time and hospital stay, more significantly reduced inflammatory factor levels, and did not increase the incidence of adverse reactions. |
| Yang et al. (2025) | 30 | 30 | 33 | 27 | T:38.71 ± 3.21  C: 38.67 ± 3.18 | TRQ 20 mL Qd + CM | CM (antiviral drugs and so on) | 5 | a, b, c, g, h, f | TRQ + CM significantly improved clinical efficacy, accelerated the resolution of symptoms such as fever, cough, wheezing, and rales, without increasing the incidence of adverse reactions. |

Note: T: treatment; C: control; M: male; F: female; RDN: Reduning Injection; XYP: Xiyanping Injection; YHN: Yanhuning Injection; TRQ: Tanreqing Injection; XBJ: Xuebijing Injection; SHL: Shuanghuanglian Injection; QKL: Qingkailing Injection; CM: conventional medicine; a: Total clinical effective rate; b: Antipyretic time; c: Cough disappearance time; d: TNF-α level; e: IL-6 level; f: Incidence of adverse reactions; g: Disappearance time of lung rales; h: Duration of asthma; i: Hospitalization time; j: CD4 level; k: CD4/CD8 ratio; l: IgM level; m: IgG level; n: IL-8 level; o: hs-CRP level.
