# Supplementary figures and images for: Therapeutic effects of traditional Chinese medicine injections with heat-clearing and detoxifying properties on viral pneumonia: a systematic review and network meta-analysis
Source: Front Pharmacol. 2026 May 14;17:1771777. doi: 10.3389/fphar.2026.1771777 (PMC13216718; doi:10.3389/fphar.2026.1771777)

**Supplementary Material 7: ConPhyMP-checklists**

**
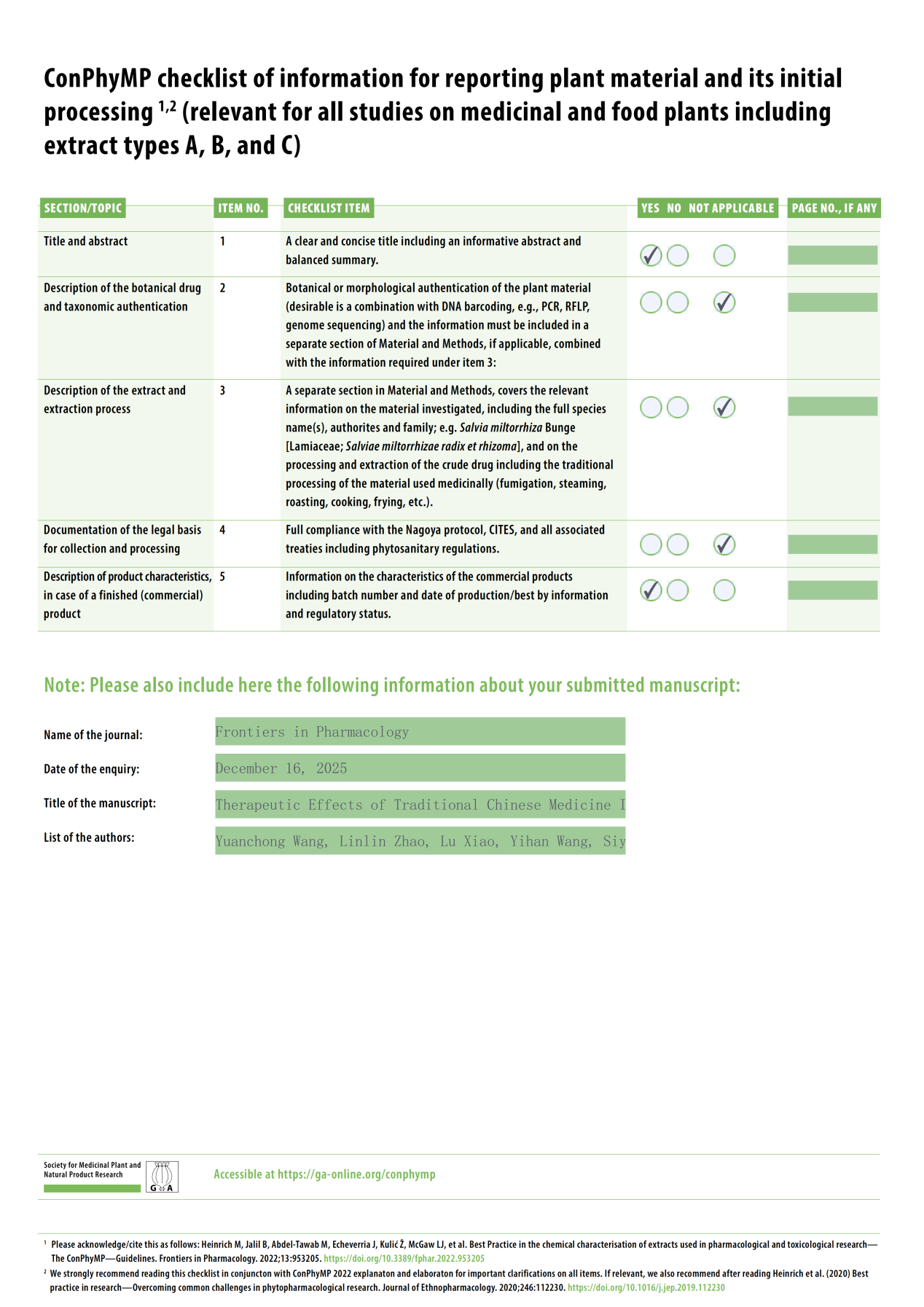

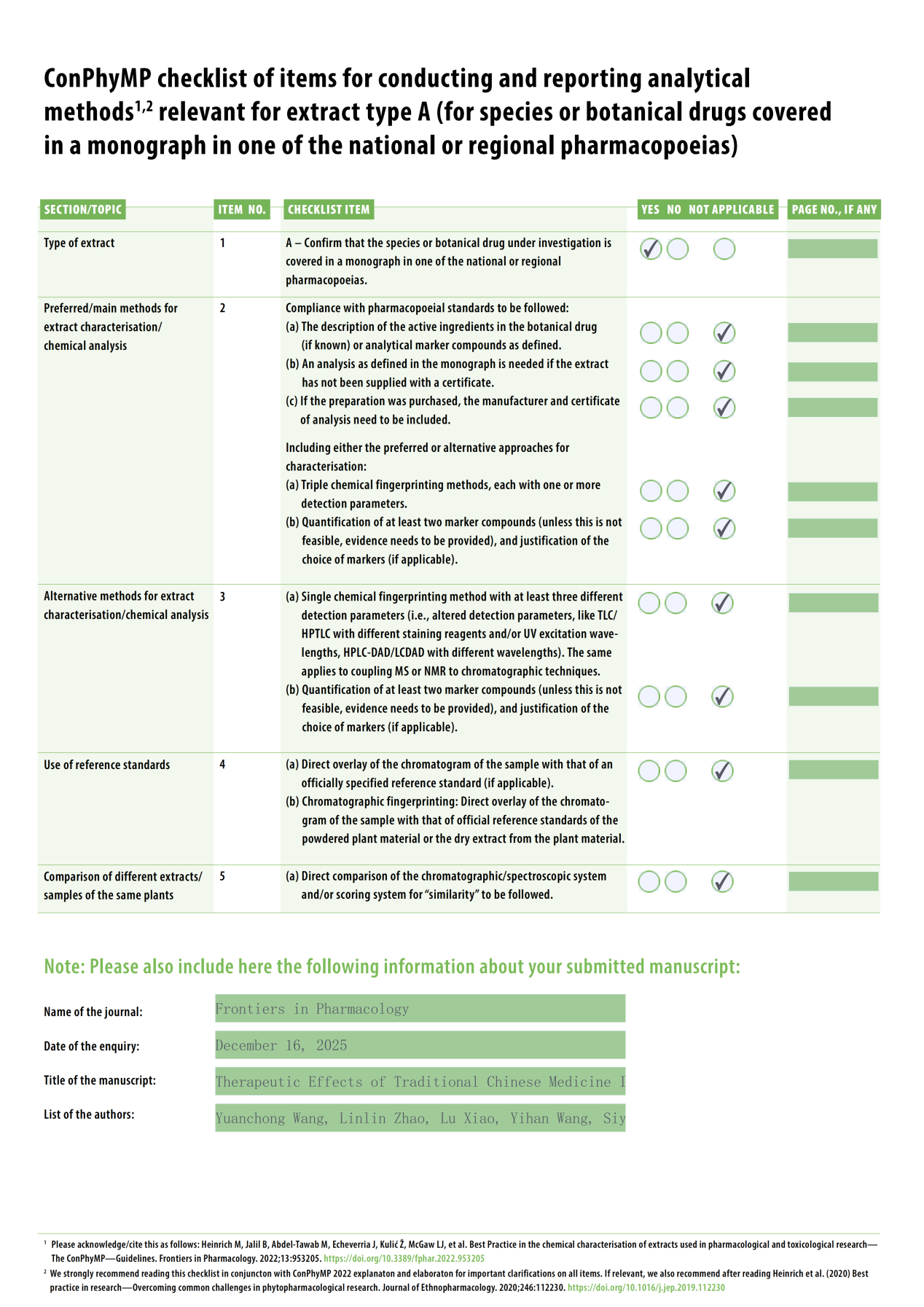
**

Supplement: Supplementary file 5 [file Supplementaryfile7.docx]
