# Supplementary material for: Therapeutic effects of traditional Chinese medicine injections with heat-clearing and detoxifying properties on viral pneumonia: a systematic review and network meta-analysis
Source: Front Pharmacol. 2026 May 14;17:1771777. doi: 10.3389/fphar.2026.1771777 (PMC13216718; doi:10.3389/fphar.2026.1771777)
Supplement: Supplementary file 6 [file Supplementaryfile8.docx]

**Supplementary Material 8: R Code for Network Meta-Analysis**

library(BUGSnet)

library(netmeta)

library(readr)

data_bugs <- data.prep(arm.data = sim_data,

varname.t = "treatment",

varname.s = "study")

net.plot(data_bugs,

node.scale = 0.6,

edge.scale = 0.6,

label.offset1 = 1.2,

study.counts = TRUE)

network.char <- net.tab(data = data_bugs,

outcome = "mean",

N = "n",

type.outcome = "continuous")

print(network.char)

fixed_model <- nma.model(data = data_bugs,

outcome = "mean",

N = "n", sd = "sd",

reference = "CM",

family = "normal",

link = "identity",

effects = "fixed")

fixed_results <- nma.run(fixed_model, n.adapt = 20000, n.iter = 50000)

random_model <- nma.model(data = data_bugs,

outcome = "mean",

N = "n", sd = "sd",

reference = "CM",

family = "normal",

link = "identity",

effects = "random")

random_results <- nma.run(random_model, n.adapt = 20000, n.iter = 50000)

pdf("model_fit.pdf", width = 10, height = 5)

par(mfrow = c(1,2))

nma.fit(fixed_results, main = "Fixed Effect Model")

nma.fit(random_results, main = "Random Effects Model")

dev.off()

consistency_model <- nma.model(data = data_bugs,

outcome = "mean",

N = "n", sd = "sd",

reference = "CM",

family = "normal",

link = "identity",

type = "consistency",

effects = "random")

consistency_results <- nma.run(consistency_model,

n.adapt = 20000,

n.burnin = 4000, # Adjusted from original 4 to a sensible value

n.iter = 50000)

pdf("forest_plot.pdf")

nma.forest(nma = consistency_results,

log.scale = FALSE,

central.tdcy = "mean",

comparator = "CM")

dev.off()

pdf("rankogram.pdf")

sucra.out <- nma.rank(consistency_results,

largerbetter = FALSE,

sucra.palette = "Set1")

print(sucra.out$rankogram)

print(sucra.out$sucraplot)

print(sucra.out$sucratable)

dev.off()

league.out <- nma.league(consistency_results,

central.tdcy = "mean",

log.scale = FALSE)

pdf("league_heat.pdf", width = 10, height = 6)

league.out$heatplot

dev.off()

nmadata_pair <- pairwise(treatment = treatment,

mean = mean, sd = sd, n = n,

studlab = study,

data = sim_data)

m.netmeta <- netmeta(TE = TE,

seTE = seTE,

treat1 = treat1,

treat2 = treat2,

studlab = studlab,

data = nmadata_pair,

sm = "MD",

fixed = FALSE,

random = TRUE,

reference.group = "CM",

details.chkmultiarm = TRUE)

summary(m.netmeta)

treat_order <- c("DrugA", "DrugB", "DrugC", "CM")

colors <- c("DrugA" = "goldenrod4", "DrugB" = "pink",

"DrugC" = "orange3", "CM" = "yellow3")

pdf("funnel_plot.pdf", width = 8, height = 6)

funnel(m.netmeta,

order = treat_order,

pch = rep(19, length(treat_order)),

col = colors[treat_order],

pooled = "random",

pos.legend = "topleft",

method.bias = "Egger",

cex.legend = 0.7)

dev.off()
