# Supplementary material for: Therapeutic effects of traditional Chinese medicine injections with heat-clearing and detoxifying properties on viral pneumonia: a systematic review and network meta-analysis
Source: Front Pharmacol. 2026 May 14;17:1771777. doi: 10.3389/fphar.2026.1771777 (PMC13216718; doi:10.3389/fphar.2026.1771777)
Supplement: Supplementary file 7 [file Supplementaryfile2.docx]

**Supplementary Material 2: Search strategy**

| **Search strategy** | **Results** | |
| --- | --- | --- |
| **PubMed**  (((("Injections"[MeSH] OR "Injection"[Title/Abstract] OR "Injectables"[Title/Abstract] OR "Injectable"[Title/Abstract])) OR (((Medicine, Chinese Traditional[MeSH Terms]) OR (Zhong Yi Xue[Title/Abstract]) OR (Chung I Hsueh[Title/Abstract]) OR (Hsueh, Chung I[Title/Abstract]) OR (Traditional Medicine, Chinese[Title/Abstract]) OR (Chinese Traditional Medicine[Title/Abstract]) OR (Traditional Chinese Medicine[Title/Abstract]) OR (Chinese Medicine, Traditional[Title/Abstract]) OR (Traditional Tongue Diagnosis[Title/Abstract]) OR (Tongue Diagnoses, Traditional[Title/Abstract]) OR (Tongue Diagnosis, Traditional[Title/Abstract]) OR (Traditional Tongue Diagnoses[Title/Abstract]) OR (Traditional Tongue Assessment[Title/Abstract]) OR (Tongue Assessment, Traditional[Title/Abstract]) OR (Traditional Tongue Assessments[Title/Abstract])))) OR (("Drugs, Chinese Herbal"[MeSH] OR "Chinese Herbal Drugs"[Title/Abstract] OR "Chinese Plant Extracts"[Title/Abstract]))) AND (((Pneumonia, Viral[MeSH Terms]) OR (Pneumonias, Viral[Title/Abstract]) OR (Viral Pneumonia[Title/Abstract]) OR (Viral Pneumonias[Title/Abstract]))) AND (1948:2025[pdat])  The search was conducted from the establishment of the library to December 12, 2025, and 3125 results were retrieved. | 3125 | |
| **WOS**  (((TS=("Injections" OR "Injection" OR "Injectables OR Injectable")) OR TS=(("Medicine, Chinese Traditional") OR ("Zhong Yi Xue") OR ("Chung I Hsueh") OR ("Hsueh, Chung I") OR ("Traditional Medicine, Chinese") OR ("Chinese Traditional Medicine") OR ("Traditional Chinese Medicine") OR ("Chinese Medicine, Traditional") OR ("Traditional Tongue Diagnosis") OR ("Tongue Diagnoses, Traditional") OR ("Tongue Diagnosis, Traditional") OR ("Traditional Tongue Diagnoses") OR ("Traditional Tongue Assessment") OR ("Tongue Assessment, Traditional") OR ("Traditional Tongue Assessments"))) OR TS=(("Drugs, Chinese Herbal" OR "Chinese Herbal Drugs" OR "Chinese Plant Extracts"))) AND TS=(("Pneumonia, Viral") OR ("Pneumonias, Viral") OR ("Viral Pneumonia") OR ("Viral Pneumonias"))  The search was conducted from the establishment of the library to December 12, 2025, and 736 results were retrieved. | 736 | |
| **Cochrane Library**  #1: MeSH descriptor: [Injections] explode all trees  #2: (Injection):ti,ab,kw  #3: (Injectables):ti,ab,kw  #4: (Injectable):ti,ab,kw  #5: MeSH descriptor: [Medicine, Chinese Traditional] explode all trees  #6: (Zhong Yi Xue):ti,ab,kw  #7: (Chung I Hsueh):ti,ab,kw  #8: (Hsueh, Chung I):ti,ab,kw  #9: (Traditional Medicine, Chinese):ti,ab,kw  #10: (Chinese Traditional Medicine):ti,ab,kw  #11: (Traditional Chinese Medicine):ti,ab,kw  #12: (Chinese Medicine, Traditional):ti,ab,kw  #13: (Traditional Tongue Diagnosis):ti,ab,kw  #14: (Tongue Diagnoses, Traditional):ti,ab,kw  #15: (Tongue Diagnosis, Traditional):ti,ab,kw  #16: (Traditional Tongue Diagnoses):ti,ab,kw  #17: (Traditional Tongue Assessment):ti,ab,kw  #18: (Tongue Assessment, Traditional):ti,ab,kw  #19: (Traditional Tongue Assessments):ti,ab,kw  #20: MeSH descriptor: [Drugs, Chinese Herbal] explode all trees  #21: (Chinese Herbal Drugs):ti,ab,kw  #22: (Chinese Plant Extracts):ti,ab,kw  #23: MeSH descriptor: [Pneumonia, Viral] explode all trees  #24: (Pneumonias, Viral):ti,ab,kw  #25: (Viral Pneumonia):ti,ab,kw  #26: (Viral Pneumonias):ti,ab,kw  #27: #1 OR #2 OR #3 OR #4  #28: #5 OR #6 OR #7 OR #8 OR #9 OR #10 OR #11 OR #11 OR #12 OR #13 OR #14 OR #15 OR #16 OR #17 OR #18 OR #19  #29: #20 OR #21 OR #22  #30: #23 OR #24 OR #25 OR #26  #31: #27 OR #28 OR #29  #32: #30 AND #31  The search was conducted from the establishment of the library to December 12, 2025, and 573 results were retrieved. | 573 | |
| **Embase**  ('injections'/exp OR 'injection':ti,ab OR 'injectables':ti,ab OR 'injectable':ti,ab OR 'medicine, chinese traditional'/exp OR 'zhong yi xue':ti,ab OR 'chung i hsueh':ti,ab OR 'hsueh, chung i':ti,ab OR 'traditional medicine, chinese':ti,ab OR 'chinese traditional medicine':ti,ab OR 'traditional chinese medicine':ti,ab OR 'chinese medicine, traditional':ti,ab OR 'traditional tongue diagnosis':ti,ab OR 'tongue diagnoses, traditional':ti,ab OR 'tongue diagnosis, traditional':ti,ab OR 'traditional tongue diagnoses':ti,ab OR 'traditional tongue assessment':ti,ab OR 'tongue assessment, traditional':ti,ab OR 'traditional tongue assessments':ti,ab OR 'drugs, chinese herbal'/exp OR 'chinese herbal drugs':ti,ab OR 'chinese plant extracts':ti,ab) AND ('pneumonia, viral'/exp OR 'pneumonias, viral':ti,ab OR 'viral pneumonia':ti,ab OR 'viral pneumonias':ti,ab) AND [<1966-2025]/py  The search was conducted from the establishment of the library to December 12, 2025, and 880 results were retrieved. | 880 | |
| **CNKI**  (SU = 'Pneumonia, Viral' OR SU = 'Viral Pneumonia') AND (SU = 'Injections' OR SU = 'Injectables' OR SU = 'Plant Preparations' OR SU = 'Herbal Preparations' OR SU = 'Herbal Medicine' OR SU = 'Plant Extracts' OR SU = 'Traditional Chinese Medicine' OR SU = 'Extracts' OR SU = 'Injectables' OR SU = 'Traditional Chinese Medicine Injections')  The search was conducted from the establishment of the library to December 12, 2025, and 487 results were retrieved. | 487 | |
| **WanFang**  SU=("Pneumonia, Viral" OR "Viral Pneumonia") AND SU=("Injections" OR "Injectables" OR "Liquid Injections" OR "Plant Preparations" OR "Herbal Preparations" OR "Herbal Medicine" OR "Plant Extracts" OR "Traditional Chinese Medicine" OR "TCM" OR "Extracts" OR "Injectables" OR "Traditional Chinese Medicine Injections" OR "TCM Injections")  The search was conducted from the establishment of the library to December 12, 2025, and 699 results were retrieved. | 699 |  |
| **VIP**  (M=Pneumonia, Viral OR M=Viral Pneumonia) AND (M=Injections OR M=Injectables OR M=Phytotherapeutic Drugs OR M=Drugs, Chinese Herbal OR M=Chinese Herbal Drugs OR M=Plant Extracts, Medicinal OR M=Medicine, Chinese Traditional OR M=Plant Extracts OR M=Injections, Herbal OR M=Herbal Injections)  The search was conducted from the establishment of the library to December 12, 2025, and 359 results were retrieved. | 359 |  |
| **CBM**  ("viral pneumonia"[Common Fields] OR ("pneumonia"[Common Fields] AND "viral"[Common Fields])) AND ("injections"[Common Fields] OR "liquid injections"[Common Fields] OR "plant preparations"[Common Fields] OR "herbal preparations"[Common Fields] OR "herbal medicine"[Common Fields] OR "plant extracts"[Common Fields] OR "traditional chinese medicine"[Common Fields] OR "extracts"[Common Fields] OR "injectables"[Common Fields] OR "TCM injections"[Common Fields])  The search was conducted from the establishment of the library to December 12, 2025, and 992 results were retrieved. | 992 |  |
