# Supplementary material for: Therapeutic effects of traditional Chinese medicine injections with heat-clearing and detoxifying properties on viral pneumonia: a systematic review and network meta-analysis
Source: Front Pharmacol. 2026 May 14;17:1771777. doi: 10.3389/fphar.2026.1771777 (PMC13216718; doi:10.3389/fphar.2026.1771777)
Supplement: Supplementary file 8 [file Supplementaryfile4.docx]

## Supplementary Material 4

## 1 Comparison of DIC and I^2^ under different models

| NO. | Outcome | Fixed effects model | Random effects model | Model selection |
| --- | --- | --- | --- | --- |
| 1 | Total clinical effective rate | DIC=160.93  I^2^=0 | DIC=162  I^2^=0 | Fixed effects model |
| 2 | Incidence of adverse reactions | DIC=84.94  I^2^=0 | DIC=87.02  I^2^=0 | Fixed effects model |
| 3 | Antipyretic time | DIC=783.9  I^2^=85% | DIC=211.43  I^2^=2% | Random effects model |
| 4 | Cough disappearance time | DIC=492.39  I^2^=78% | DIC=187.7  I^2^=3% | Random effects model |
| 5 | TNF-α level | DIC=346.91  I^2^=91% | DIC=63.88  I^2^=4% | Random effects model |
| 6 | IL-6 level | DIC=242.2  I^2^=88% | DIC=56.34  I^2^=5% | Random effects model |
| 7 | Disappearance time of lung rales | DIC=614.96  I^2^=85% | DIC=172.12  I^2^=1% | Random effects model |
| 8 | Duration of asthma | DIC=279.34  I^2^=83% | DIC=86.26  I^2^=2% | Random effects model |
| 9 | Hospitalization time | DIC=626.05  I^2^=95% | DIC=59.62  I^2^=4% | Random effects model |
| 10 | CD4 level | DIC=61.05  I^2^=78% | DIC=24.07  I^2^=9% | Random effects model |
| 11 | CD4/CD8 ratio | DIC=46.05  I^2^=45% | DIC=36.95  I^2^=9% | Random effects model |
| 12 | IgM level | DIC=31.25  I^2^=81% | DIC=12.04  I^2^=18% | Random effects model |
| 13 | IgG level | DIC=21.55  I^2^=70% | DIC=12.17  I^2^=19% | Random effects model |
| 14 | IL-8 level | DIC=64.53  I^2^=80% | DIC=24.03  I^2^=9% | Random effects model |
| 15 | hs-CRP level | DIC=49.93  I^2^=53% | DIC=36.31  I^2^=8% | Random effects model |

## 2 Assessment of the transitivity hypothesis

As shown in the box plot, the distribution of sample sizes in different TCMIs is roughly similar, suggesting that the treatment groups have a certain degree of comparability in terms of research scale.

**3	Sensitivity analysis**

**3.1	Total clinical effective rate**

**3.2	Antipyretic time**

**3.3	Cough disappearance time**

**3.4	TNF-α level**

**3.5	IL-6 level**

**3.6	Incidence of adverse reactions**

**3.7	Disappearance time of lung rales**

**3.8	Duration of asthma**

**3.9	Hospitalization time**

**3.10	CD4 level**

**3.11	CD4/CD8 ratio**

**3.12	IgM level**

**3.13	IgG level**

**3.14	IL-8 level**

3.15 **hs-CRP level**

## 4 Subgroup analysis

## 4.1 Total clinical effective rate

## 4.1.1 Aged under 18 years

**4.1.2	Aged 18 years or older**

## 4.2 Antipyretic time

## 4.2.1 Aged under 18 years

**4.2.2	Aged 18 years or older**

**4.3	Cough disappearance time**

**4.3.1	Aged under 18 years**

**4.3.2	Aged 18 years or older**

**4.4	TNF-α level**

**4.4.1	Aged under 18 years**

**4.4.2	Aged 18 years or older**

**4.4.3	Duration under 7 days**

**4.4.4	Duration of 7 days or more**

**4.4.5	Duration unknown**

**4.5	IL-6 level**

**4.5.1	Aged under 18 years**

**4.5.2	Aged 18 years or older**

**4.5.3	Duration under 7 days**

**4.5.4	Duration of 7 days or more**

## 4.5.5 Duration unknown

## 4.6 Incidence of adverse reactions

**4.6.1	Aged under 18 years**

**4.6.2	Aged 18 years or older**

**4.7	Disappearance time of lung rales**

**4.7.1	Aged under 18 years**

**4.7.2	Aged 18 years or older**

**4.8	Duration of asthma**

**4.8.1	Aged under 18 years**

**4.8.2	Aged 18 years or older**

**4.9	Hospitalization time**

**4.9.1	Aged under 18 years**

**4.9.2	Aged 18 years or older**

**4.10	CD4 level**

**4.10.1	Aged under 18 years**

**4.10.2	Aged 18 years or older**

**4.11	CD4/CD8 ratio**

**4.11.1	Aged under 18 years**

**4.11.2	Aged 18 years or older**

**4.12	IgM level**

**4.12.1	Aged under 18 years**

**4.12.2	Aged 18 years or older**

**4.13	IgG level**

**4.13.1	Aged under 18 years**

**4.13.2	Aged 18 years or older**

**4.14	IL-8 level**

**4.14.1	Aged under 18 years**

**4.14.2	Aged 18 years or older**

**4.15	hs-CRP level**

**4.15.1	Aged under 18 years**

**4.15.2	Aged 18 years or older**
